# End-of-Life Care: A Multimodal and Comprehensive Curriculum for Graduating Medical Students Utilizing Experiential Learning Opportunities

**DOI:** 10.15766/mep_2374-8265.11149

**Published:** 2021-04-27

**Authors:** Justin M. Jeffers, Sharon Bord, Jody E. Hooper, Carol Fleishman, Danelle Cayea, Brian Garibaldi

**Affiliations:** 1 Assistant Professor, Department of Pediatrics, Division of Pediatric Emergency Medicine, Johns Hopkins University School of Medicine; 2 Assistant Professor, Department of Emergency Medicine, Johns Hopkins University School of Medicine; Co-Director, Transition to Residency, Internship, and Preparation for Life Events (TRIPLE); 3 Associate Professor and Director of Autopsy, Department of Pathology and Oncology, Johns Hopkins University School of Medicine; 4 Senior Simulation Educator, Johns Hopkins Medical Simulation; Standardized Patient Lead, TRIPLE; 5 Associate Professor, Department of Medicine, Johns Hopkins University School of Medicine; 6 Associate Professor, Department of Medicine and Physiology, Pulmonary and Critical Care, Johns Hopkins University School of Medicine; Co-Director, TRIPLE

**Keywords:** Palliative Care, End-of-Life Care, Standardized Patients, Simulation, Critical Care

## Abstract

**Introduction:**

End-of-life (EOL) care is an essential skill for most physicians and health care providers, yet there continues to be an educational gap in medical education literature for these skills. The Johns Hopkins School of Medicine developed the Transition to Residency, Internship, and Preparation for Life Events (TRIPLE) curriculum with the primary goal of preparing graduating medical students for life after medical school.

**Methods:**

The EOL module was one of many within the TRIPLE curriculum and consisted of two half-day sessions that targeted EOL care, death, dying, and communication skills. The first half-day session focused on a standardized patient encounter where learners initiated and completed an EOL care goals conversation around a living will. The second half-day session focused on death and dying. It included didactic sessions on organ donation, autopsy/death certificates, a simulation-based learning session on ending a resuscitation, and a standardized patient encounter where learners disclosed the death of a loved one. End-of-day and end-of-course evaluations were collected via anonymous online surveys.

**Results:**

In 2019, 120 students and 26 instructors participated in TRIPLE. Students rated the EOL module overall as 4.6 of 5 (*SD =* 0.6) and rated instructors overall as 4.6 of 5 (*SD =* 0.6).

**Discussion:**

By implementing a thorough and diverse curriculum with a variety of modalities and targeted skills, learners may be better prepared to care for patients dealing with EOL care issues. Further, the generalization of these skills may assist learners in a variety of other aspects of patient and family care.

## Educational Objectives

By the end of this activity, learners will be able to:

1. Describe key differences among advance care planning, living wills, durable power of attorney, and do-not-resuscitate orders.
2. Demonstrate a focus on a patient's values and goals (rather than on treatment options) for advance care planning through a conversation with a patient about preferences for life-sustaining treatment, while using appropriate elements of informed consent.
3. Describe emotional and communication considerations unique to end-of-life discussions with parents and children.
4. Demonstrate the proper method of pronouncing a person to be dead and inform a family member of the death in a clear and compassionate manner.
5. Correctly complete a death certificate as well as understand reasons and procedure for autopsy.
6. Describe appropriate approaches to discussing organ donation with families.

## Introduction

Palliative and end-of-life (EOL) care first received consideration in medical school education in the late 1960s^1^ and in 2000, the Liaison Committee on Medical Education developed a directive focusing on EOL care.^2^ Despite 50 years of recognized importance, the literature continues to highlight the inadequacy of EOL and palliative care training.^3–6^ To highlight this, a 2016 paper by Schmit, et al. showed that over half of all medical students receive little to no classroom EOL training and only roughly 4% of medical students had participated in more than 10 EOL conversations.^7^ This inadequacy becomes even more relevant when one considers the current aging population and prevalence of chronic illness. In the United States more than 90% of persons over the age of 65 have one or more chronic diseases, and 10% of children have a chronic condition, necessitating EOL and goals-of-care conversations.^8,9^

Although there are numerous published curricula for undergraduate medical education, two review articles revealed a lack of consistency, meaningful learning, and satisfaction with EOL and palliative care learning.^2,5^ Most curricula, both undergraduate and graduate, only focus on a single aspect of EOL and palliative care such as ethical dilemmas, communication around advanced directives, or delivering challenging news.^10,11^ Other curricula only utilize a single educational modality such as lecture, small-group discussions, computer-based modules, standardized patients (SPs), or simulation-based learning (SBL).^10,12–15^ Lastly, other curricula are specific to singular clinical situations or patient populations making them hard to generalize to other clinical environments.^12,16,17^

This curriculum is unique to the literature in that it provides a multiprofessional, multiaspect, and multimodal experiential learning opportunity for graduating medical students around EOL goals, palliative care, and death. The skills learned may be generalizable to other areas of practice as well. The curriculum utilizes a combination of didactic sessions along with SP encounters and SBL strategies in order to deliver content with a higher degree of interaction while aligning with the medical school's overall approach to promoting professional identity development and scientific inquiry.^18^

The curriculum was delivered during the Johns Hopkins University School of Medicine's Transition to Residency, Internship, and Preparation for Life Events (TRIPLE) course for graduating medical students. TRIPLE is a 2-week required course focusing on core skills to prepare students to succeed as they graduate medical school, including modules in financial planning and stress management to advanced life support and rapid response training. The course is run twice each spring to accommodate all graduating medical students.

## Methods

### Overview

Although the entire EOL module could occur in 1 day, we purposefully split it into 2 half-days (EOL 1 and EOL 2). This was done due to the large amount of content delivered, the heavy nature of the content, and to allow flexibility with room/equipment turnover. Prior to the module, we gave the students a small amount of required reading.^19–21^ EOL 1 focused on a small-group SP encounter in which learners had a conversation around EOL care and advanced directives with a patient who had a progressing chronic disease. EOL 2 focused on death and dying. Learners participated in small-group didactic sessions about organ donation and an interactive didactic session about death certificates and autopsy. EOL 2 also included an SBL activity where learners managed a cardiac arrest case, pronounced death, practiced the death exam, and finished with an SP encounter where the learner disclosed the patient's death to a loved one.

Overall classroom, personnel, and equipment needs will be institutionally specific depending on the number of learners. For the purpose of this module, needs will be illustrated based on approximately 60 learners.

### EOL 1

#### Preparation

We developed EOL 1 to target Educational Objectives 1, 2, and 3. It was an approximately 3-hour session that required one SP and one faculty preceptor per small group of four to six learners. Prior to the session, we grouped students based on future specialty, and when possible paired them with volunteer faculty from the same specialty. Faculty guides are presented in Appendix A. In an effort to make this session more meaningful and applicable, students entering into pediatrics residency training participated in a pediatric case (Appendix B) and students entering adult patient specialties were given an adult case (Appendix B). Within the student handout was a patient summary as well as their specific task for this exercise.

#### Setting

We held this module in an SP room. However, it could be done in any available space. Ideal setup included two entry points to the room: one for the SP and one for the learner group to maintain appropriate separation and SP realism. Setup also included an exam table and/or chair for the SP as well as a chair for the learner who had the conversation, chair for the faculty member, and chairs for the rest of the learner group. Chairs for faculty and the rest of the learner group were placed such that they could see and hear the interaction, but were clearly apart from the primary learner and the SP.

#### Encounter

To encourage local customization and flexibility, we included an opportunity to have a 30-minute didactic session specific to the institution prior to the SP encounter. At our institution, we invited palliative care faculty to discuss a topic of their choice. Previous didactic sessions included topics on research in palliative care, history of palliative care, how to have challenging conversations, and a general overview of goals-of-care conversations. This component was not mandatory to the module, but served as a way to further include palliative care faculty. If institutions decide to utilize this time, appropriate audiovisual presentation technology is required.

We provided learners with their handouts (Appendix B) prior to the session via an online learning and course management platform. Learners were split into groups of four to six. Typically, the groups were four or five learners unless there was an extra learner for a given specialty. We chose to have a larger learner group with same or similar specialties rather than have one learner be an outlier in another group. Learners then transitioned to their SP room with their assigned faculty. Faculty introduced themselves and outlined the plan for the session. Appendix A includes the general timeline. During the session, the group of learners functioned as one team initiating a goals-of-care conversation. Each learner had approximately 7–10 minutes to progress the conversation, and when the SP left the room after each segment, the faculty preceptor provided approximately 5 minutes of feedback. The other learners in the room were also expected to offer feedback and insights to each other. Once each learner had taken part in the patient conversation and the final faculty feedback had occurred, the SP returned and provided approximately 15 minutes of feedback to the group.

#### SP recruitment/training

We recruited SPs from our institution's SP database. SP materials are provided in Appendix C. The SPs attended a 3-hour training for each SP exercise. If they were not experienced with preparing and providing feedback, they also attended a 3-hour training specifically about feedback skills. The SP training occurred 1 week before the first date an activity was held. Faculty were encouraged to join the training sessions to speak with the SPs about the clinical aspects of the case. The faculty may also guide the SPs in how patients in these situations appear/behave. The SPs were provided with the case materials, feedback topic guides, and the logistics of the exercise. We also gave the SPs the student handouts and the faculty guides to promote consistency. During the training session, SPs were guided through the case using the training materials provided in Appendix C. The session was led by experienced SPs as well as faculty (Carol Fleishman). Additionally, the SPs watched videos of encounters from previous years, providing an opportunity to see examples of how the students interacted with the SPs and to practice preparing feedback specific to the encounters they observed. These videos are not included and should be made anew when implemented at other institutions.

#### Learner assessment

Learner assessment was formative in nature and was provided by both the faculty member and the SP.

### EOL 2 Overview

EOL 2 was approximately 4 hours and focused on managing death and disclosure of death conversations. It required two classrooms capable of seating up to 30 students with audiovisual presentation capabilities. Additionally, three SBL rooms and adult size manikins were required. To promote realism, high-fidelity manikins were recommended but not required. Ideally two faculty per SBL room were needed: one who operated the manikin and one who participated and debriefed the SBL scenario. SPs participated as the patient's family member during the disclosure of death conversation. Ideally, the disclosure conversation occurred in an individual and private room, such as a clinic room, but any location can be made to work with adequate spacing, privacy screens, or similar.

After a brief 5-minute introduction and welcome, we split the learners into three groups for the three stations: two didactic sessions and one SBL/SP activity. The didactic sessions focused on autopsy procedures and completing death certificates (Station I), and organ donation (Station II). We allotted 75 minutes for all three stations with a 15-minute break built into the organ donation session.

### EOL 2 Station I

This didactic session focused on the role of the medical examiner, completing a death certificate, and appropriately determining cause of death. The presentation and notes are included in Appendices D and E, respectively. Instruction by a knowledgeable pathologist was helpful. This was an interactive didactic session where learners acquired skills to accurately and appropriately complete a death certificate. Learners also received instruction on basic autopsy laws using real examples as well as interactive cases, as well as how to approach a family regarding autopsy. It is important to note that local and state laws and regulations may differ from those discussed.

### EOL 2 Station II

This didactic session focused on organ donation. This was a 60-minute presentation with a 15-minute break immediately after for the learners. Because local agencies, laws, and regulations around organ donation vary, we have intentionally not included the presentation used at our institution. Objectives for the organ donation session included: (1) describe appropriate approaches to discussing organ donation with families; (2) understand local organ donation agencies and procedures; (3) describe the need and importance of increasing organ donation; (4) understand your role as a physician in the process of organ donation. We encourage users of this curriculum to engage with local resources in order to provide the most accurate and valuable content to their learners.

### EOL 2 Station III

#### Preparation

The EOL 2 SBL and SP activity were performed in the Johns Hopkins Outpatient Center Simulation Center at Johns Hopkins University. The activity was developed to target Educational Objective 4: Demonstrate the proper method of pronouncing a person to be dead and inform a family member of the death in a clear and compassionate manner. SP preparation was similar to EOL 1 as described above. The primary differences were: (1) format − the EOL 2 SP encounter was a 1:1 encounter with no faculty facilitator, (2) duration − the EOL 2 SP encounter was 25 minutes total, and (3) the SPs were provided with Appendix H during their training.

#### Overview

For station III, we split the group into three subgroups, giving each of the three identical SBL set-ups six to eight learners. Learners began with the SBL case of a brief asystolic arrest (Appendix F). Faculty then debriefed the case medically and discussed how to perform a death exam (Appendix G). The goal was to focus on the death exam and not to spend significant time on the management of asystole (learners received advanced cardiac life support, ACLS, instruction during other TRIPLE modules). If implemented with learners who do not have ACLS instruction, the case can be modified to minimize ACLS performance. For example, learners may enter the room as the resuscitation is ending, or the faculty may participate as a physician lead running the event and guide the learners through the resuscitation.

Learners were then given opportunities to practice performing a death exam. Next, learners transitioned to a 1:1 SP station without faculty participation (only SP interaction and feedback; Appendix H). Appendix G also includes a full timeline to assist with organization and rotation of learners.

#### SBL equipment

We recommend the following equipment for the successful implementation of the simulation case:

• A high-fidelity manikin with software allowing for programing a cardiac arrest event.
• Patient monitor able to display cardiac rate and rhythm, pulse oximetry, respiratory rate, noninvasive blood pressure, and end tidal CO2.
• Cardiac monitoring equipment, pulse oximetry, and noninvasive blood pressure cuff.
• Intraosseous access device and/or peripheral intravenous access supplies.
• Oxygen delivery devices such as nasal cannula, nonrebreather, and bag-valve mask device.
• Syringes and needles of various sizes.
• Vials marked epinephrine 1:10,000 and 1 liter bags of normal saline.
• Adult code cart, defibrillator, and chest compression adjuncts such as stepstool and backboard.

#### SBL personnel

Although the simulation can be done with only one facilitator, we found that having two facilitators led to a subjectively better experience for the facilitators. One facilitator operated the manikin from the control room and the other served as a nurse confederate during the simulation as well as the primary debriefer of the event. The nurse confederate provided patient details when asked and served as a competent nurse during the case.

#### SBL implementation

Immediately prior to the start of the case, faculty told learners they were a part of the ICU code response team and that they were being called to an adult general inpatient floor. Faculty then provided learners with a sign-out sheet, recent laboratory values, as well as a recent progress note (Appendix F). Learners were given about 2 minutes to read over the progress notes. Upon entering the room, the nurse confederate told the learners that the patient was in asystole and needed assistance. The learners were also told they can use whatever resources at their disposal such as websites, mobile phone applications, or information cards. The manikin operator was responsible for starting and stopping the case based on learner entry to the room, and when the nurse confederate stopped the case. The operator also brought up the patient monitor data when asked. The operator did need to be able to see and hear what was taking place in the simulation room, but there was no routine need for communication between the control room and the simulation room.

#### SBL assessment/debreif

Assessment was formative and was based on learner performance during the case. This occurred during the debriefing, which lasted 15–20 minutes. The learner handout, a timing flow chart, and key teaching and debriefing points on performing a death exam are included in the faculty guide (Appendix G). Little time was spent focusing on the medical management and should target glaring medical errors in the management of asystole. The primary purpose of the debriefing time was to educate learners about the death exam and to give learners the opportunity to practice performing a death exam in a safe learning environment. At the end of the debriefing, facilitators spent up to 10 minutes setting the stage for the upcoming SP encounter. During this time, the facilitator described where to go as well as provide any suggestions they may have for navigating the conversation.

#### SP setting

Ideally the EOL 2 SP encounter would occur in a private room with one chair for the SP and one chair for the learner. If this is not an option, any room or space will suffice with an attempt to preserve privacy and individual encounters. There is an opportunity to be creative, if needed. For example, perhaps the SP encounter occurs via phone or video chat with an out-of-state next of kin.

#### SP encounter

The SP materials include a case summary, student instructions, as well as direct and thorough SP instructions (Appendix H). Learners were given a few minutes to arrive outside their assigned room and to gather their thoughts. When instructed, learners knocked and entered the room. They then spent about 10 minutes disclosing the death of the patient to the family member. The SP then spent 15 minutes providing feedback to the learner.

#### SP assessment

Assessment was formative and occurred during the last 15 minutes of the encounter. The assessment tool used for feedback is included in Appendix H.

### End-of-Course Evaluation

When implementing higher emotional SP activities, it is important to provide resources and opportunities to care for the SPs. For both SP encounters, we provided at least 15 minutes (more if needed) additional time to de-role and debrief with a staff person. Additionally, SPs were scheduled such that if on the day of the activity they were not emotionally prepared to carry out the case, they could step aside. Lastly, because SPs were hospital staff, they had access to all the resources the hospital provided to any other staff member for dealing with emotional stress.

At the end of TRIPLE, an anonymous online survey was distributed to all learners via an online platform to evaluate the curriculum. The EOL section consisted of 19 questions (Appendix I) ranging from overall satisfaction to an open-ended question inquiring what learners felt was something they learned from the module. Closed-ended questions consisted of 5-point rating scales, with 1 (choice *a* in Appendix I) being the lowest score and 5 (choice *e* in Appendix I) being the highest. Depending on the question asked, the scales ranged from *poor* (1) to *excellent* (5) or *strongly disagree* (1) to *strongly agree* (5). *M* and *SD* were calculated for closed-ended items.

## Results

In 2019, 120 students and 26 instructors participated in TRIPLE. Of learners, 106 completed the survey (88%), but not all learners responded to all 19 questions (Table 1). Twenty-six unique faculty participated across the four total sessions (each EOL session was given twice to accommodate all students). Faculty included residents, fellows, and attendings from a variety of specialties including pediatrics, pediatric intensive care, palliative care, pathology, internal medicine, general surgery, emergency medicine, and otolaryngology.

**Table 1.**
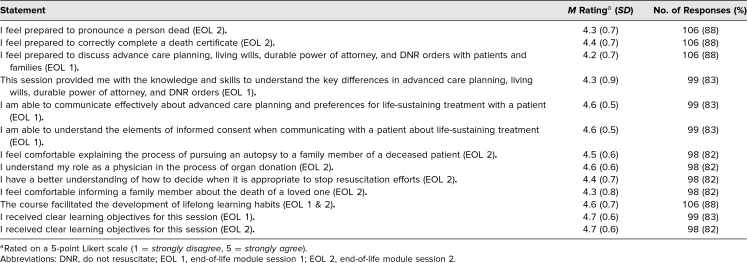
End-of-Course Evaluation Average Ratings and Response Rates for Participants (*N* = 120)

Learners who responded to the survey rated the session overall at 4.6 (*SD* = 0.6) on the *poor* (1) to *excellent* (5) Likert scale and rated the overall performance of the instructors at 4.6 (*SD* = 0.6) on a *poor* (1) to *exceptional* (5) Likert scale. When asked if the session reflected the presented educational objectives, learners rated the session at 4.6 (*SD* = 0.6) on a *strongly disagree* (1) to *strongly agree* (5) Likert scale.

Table 1 includes responses from the remaining end-of-course survey questions for which the average rating, amidst variable response rates, ranged from 4.2 (*SD* = 0.7) to 4.7 (*SD* = 0.6) on a 5-point Likert scale (1 = *strongly disagree*, 5 = *strongly agree*).

Responses to the open-ended question on what learners felt was something they learned from module were organized into themes based on relevance to EOL 1, EOL 2, as well as general comments. Overall comments were positive with many learners reiterating key module takeaways. The learners also gave suggestions for improvement, primarily focused on delineating the difference between power of attorney and living wills. Representative quotes are included in Table 2.

**Table 2.**
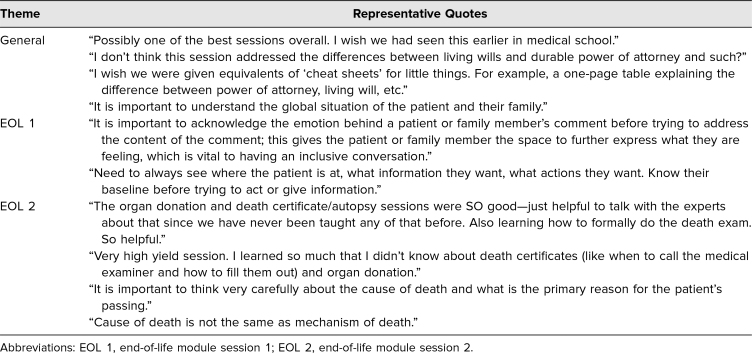
Representative Quotes From End-of-Course Evaluation Open-Ended Question “List something important you learned from this session that will help you with internship and residency.”

## Discussion

This was a curriculum designed to deliver EOL content to graduating medical students as part of the TRIPLE course. To date, this is the most robust EOL curriculum reported in the literature. The EOL module was an approximately 7-hour curriculum and consisted of multiple educational modalities including SBL, SP interactions, interactive didactic sessions, and self-guided readings. The module utilized numerous forms of active learning via visual, kinesthetic, and demonstrative instructional strategies.

Overall, the module was very well received with high learner satisfaction scores and offered to a diverse group of faculty across numerous specialties and disciplines. Although the objectives themselves were not specifically assessed, learners reported the objectives were clear and the course met those objectives. There were a few other results worth highlighting as they related to the overall objectives of this course. First, an overwhelming number of learners now feel comfortable communicating with their patients about goals-of-care and advanced planning. Second, the majority of learners will take with them lifelong learning habits as they relate to EOL care into their residency training. Lastly, learners indicated their comfort discussing autopsy with patient families as well as comfort completing death certificates. These results indicated that this curriculum filled an educational gap that continues to be described. ^22^ This curriculum addressed many of the shortcomings described in the literature,^5^ including an organized approach focusing on a diverse spectrum of objectives, skills, multidisciplinary and diverse faculty, and curricular evaluation.

The closed-ended questions had a response rate of over 80%. The open-ended questions had an approximate 15% response rate. We felt the primary contributor to this low response was evaluation fatigue. During TRIPLE, learners are asked to complete evaluations multiple times each day with a total of over 200 questions spread over the 2-week course. By the end of the 2-week course, it is understandable that a certain amount of fatigue has set in. Considering the extra effort required to type a response compared to clicking a button, this low response rate for open-ended questions is reasonable given the context.

Although provided to medical students, this curriculum has been mapped to the milestones outlined by the ACGME for hospice and palliative care medicine and can be applied to any level of learner.^23^ Some examples included addressing suffering and distress, care of the imminently dying, disease trajectory and formulation of prognosis in serious illness, patient- and family-centered communication, and complex communication around serious illness. This has value considering the lack of overreaching EOL and palliative care curricula in the literature.

Much of the delivered content can be generalized to a variety of other clinical situations adding more value to the curriculum. Learners were given an opportunity with formative feedback to practice vital communication skills and techniques. These skills were not limited to EOL care discussions. Additionally, learners were given an opportunity to manage a cardiac arrest resuscitation in an SBL environment. The benefits of this particular SBL subcurriculum have been illustrated in the literature.^24^ Lastly, the formative feedback received from SPs has been shown to improve future performance.^25–28^

There were a number of limitations to consider. The most significant revolved around the high resource needs for this curriculum. This was a faculty-intense curriculum with one SP and one faculty per four to five students for EOL 1, and six SPs and six faculty for EOL 2, in addition to didactic presenters (if they are not already faculty). Even though this can be challenging, it also provided a unique opportunity for learners to interact closely with a diverse faculty group whom they may not have had an opportunity to interact with previously. This included faculty physicians as well as fellows and residents.

Additional resource limitations included space and technology. Locations may not have the required SBL technology, audiovisual technology, or the space to accommodate a large group of learners. However, low-fidelity manikins may be substituted with minimal lost value.

One challenge faced was the diversity of experience within the learner group. By the time they reach this EOL curriculum, our learners are on the verge of graduation. They have already matched into their specialty and have likely done multiple clinical electives and rotations in their future field. Those students interested in acute care may have not been challenged by the cardiac arrest simulation. Those students interested in internal medicine or geriatrics may have not been challenged by the EOL care/goals conversation exercise. Faculty are encouraged to utilize learners past experiences to add value to the group dynamic, conversations, and learning. The students at our institution receive training on how to offer peer-to-peer feedback and were encouraged to do so throughout this curriculum. Another limitation was that of evaluation and assessment. At the time of publication, little validity evidence had been collected and analyzed around the online survey or SP evaluation tools.

Given the high emotional toll courses such as this can develop, it was important to consider specific debriefing points around mental resilience and psychological wellbeing. We intentionally did not include time for this during EOL because it was covered within other aspects of TRIPLE. Going forward, however, we will consider ways to integrate these conversations throughout EOL. Examples of this could include reinforcing the safety contract, faculty normalizing the emotional impact of this work, as well as highlighting support services.

Future directions include four main areas. First, improving the content itself. This may include reevaluating debriefing strategies used, considering how to better use technology to assist with learner retention, addressing learners concerns regarding defining terms within EOL care, and updating learner and faculty guides to promote learner performance and retention. Second, improving the evaluation and assessment of the curriculum and the learners to provide better feedback and education as well as improve the curriculum. Third, a longitudinal follow-up project to evaluate the long-term impact of this curriculum is needed. Lastly, we will continue to provide a more diverse faculty by recruiting not only faculty physicians from more subspecialties, but also social workers with EOL care expertise, as well as other advanced practice providers.

## Appendices

End-of-Life 1 Faculty Guide.docxEnd-of-Life 1 Student Handouts.docEnd-of-Life 1 Standardized Patient Materials.docxEnd-of-Life 2 PowerPoint Presentation.pptEnd-of-Life 2 Faculty Guide.docxEnd-of-Life 2 Simulation Materials.docxEnd-of-Life 2 Simulation Case Faculty Guide.docxEnd-of-Life 2 Standardized Patient Materials.docxEnd-of-Life Assessment.docx
All appendices are peer reviewed as integral parts of the Original Publication.
