# Supplementary material for: End-of-Life Care: A Multimodal and Comprehensive Curriculum for Graduating Medical Students Utilizing Experiential Learning Opportunities
Source: MedEdPORTAL. 2021 Apr 27;17:11149. doi: 10.15766/mep_2374-8265.11149 (PMC8076371; doi:10.15766/mep_2374-8265.11149)
Supplement: Supplementary file 1 — End-of-Life 1 Faculty Guide.docxEnd-of-Life 1 Student Handouts.docEnd-of-Life 1 Standardized Patient Materials.docxEnd-of-Life 2 PowerPoint Presentation.pptEnd-of-Life 2 Faculty Guide.docxEnd-of-Life 2 Simulation Materials.docxEnd-of-Life 2 Simulation Case Faculty Guide.docxEnd-of-Life 2 Standardized Patient Materials.docxEnd-of-Life Assessment.docx [file mep_2374-8265.11149-s001.zip › A. End-of-Life 1 Faculty Guide.docx]

Discussing Life-Sustaining Treatment

Adult

Faculty Guide

**Session Objectives:**

1. Demonstrate ability to establish trust and rapport *prior* to and *during* conversations about life-sustaining treatment
2. Demonstrate a focus on patient’s values and goals (rather than on treatment options) for advance care planning through a conversation with a patient about preferences for life-sustaining treatment
3. Use appropriate elements of informed consent in a conversation about life-sustaining treatment
4. Probe patients for clear understanding of meaning of preferences and goals

**Outline and Time Guideline for Session:**

| Activity | Page | Time Guideline |
| --- | --- | --- |
| I. Introductions and session set-up | 3 | 15 minutes |
| II. SP interview for practice | 4-9 | 100-110 minutes |
| III. SP feedback, wrap-up, debrief | 10 | 15 minutes |

**SECTION I: Introductions and Session Set-up**

**FACULTY INSTRUCTIONS:**

1. **Upon entering the room, please do not move the 2 chairs by the desk.** The interviewer should sit in the chair that faces the back door. The SP should sit in the chair that faces the students. It helps the students relax if they don’t have to face their peers during the interview. It also ensures the right camera angle and SP flow of traffic.
2. **Introduction:** Students will know each other and may have been in a small group together earlier in the week. Consider starting with an introduction of yourself and a very quick ice breaker (*i.e. have students introduce the person to their right and state what type of residency they are entering*). You might also consider sharing a brief story about yourself and an experience you had with discussions about life-sustaining treatment during residency or more recently that had a lasting impact on you.

Even in their 4^th^ year, students may have strong negative or positive feelings or anxiety about standardized patient activities. Probe for those and allow them to vent for a minute if needed.

1. **Session Set-up:**
   1. **Give outline of schedule**

It is expected that each student will take a turn at interviewing. There are 105 minutes available for interviewing, which gives each student about approximately 17 minutes for both interviewing and feedback.

- 1. **Remind students of basic ground rules for these types of groups.**

These should be very familiar to them, but it never hurts to explicitly state them at the beginning of a group.

- Respect different points of view and confidentiality of peers
- Be an active listener
- Feedback should be behaviorally based
- The person in the “hot-seat”, the SP, or the faculty member may call “time-out” at any time. The rest of the observers may not.

**TOTAL TIME: 15 minutes**

**SECTION II: SP Interview for Practice**

**FACULTY INSTRUCTIONS:**

1. **Ask students if they have any questions about the scenario.** They should have read the handout just prior to the session (See below). Remind them their goal as a group is to establish a “code status” for the patient and determine who their health care proxy is, but that may take several interviewers.
2. **Review the communication skills guide.** Tell students that when they are observing the interview, they may want to jot down some observations on this guide as points of feedback for their colleague. Observers should refer to this during the interview to serve as a guide for feedback. Does the interviewer follow any of the steps listed? If so, ask for specific instances and perception of the impact on the patient. Does the interviewer respond to emotions, display empathy, and build trust? Ask observers to watch for specific examples.
3. **Ask students if they have questions left over from the lecture that preceded this group or if there are specific parts of the conversation they want to talk through before inviting the SP in.** You may just talk through their questions or offer to set up a very brief role play to practice language.
4. **Ask for one student to volunteer as the first physician to interview.** *Ask the student if there is any particular skill they’d like to work on and receive feedback about. Or ask them what they think a successful encounter would look like for their brief section of the conversation in specific terms. If they say no, that is okay too for this exercise.*
5. **Bring in SP and Introduce patient.** Mr./Ms. Ward is a 55-65-year-old man/woman admitted with their 2^nd^ acute exacerbation of COPD in 9 months. Their primary care doctor introduced the idea of discussing advance directives at the last visit, but they never had a formal conversation about it prior to this hospitalization.
6. **Student interviews.** Use your judgment regarding time. One suggestion is to give 8-10 minutes for interview and ~8 minutes for feedback/debrief. Be cognizant of time constraints in an effort to give every student a chance to interview. This may require judiciously calling time-outs.
7. **Call time out.** See below. If student does not call time out before 8-10 minutes have elapsed, call time out. If the student calls time out before time is up, ask why, trouble-shoot, and give student option to rewind to just before point of frustration or they can try to recover from the stuck point.
8. **Have SP step out of room during time out between students.** This gives them a chance to jot notes for feedback. Upon resumption, this also gives you a chance to ask SP for any modifications outside of room just prior to next interviewer. When the next student is ready, you should invite the SP back in. If a student times out early, and is going to replay a part, the SP does not need to step out.
9. **Feedback**-see below for tips

**SECTION II continued**

1. **Ask for next volunteer**-Give them the option to rewind and replay or to pick up where the student left off.  *Ask if there is any particular skill they’d like to receive feedback about.* The SP will assume all interviewers are 1 doctor and there will not be a need for students to introduce themselves each time or restart relationship-building. BUT, it will be helpful to the SP if the student says their name for feedback purposes.
2. **Repeat steps 6 through 9** until all students have interviewed or time is up. **DO NOT** have each student restart the scenario from the beginning.
3. **IF YOU COMPLETE THE INTERVIEW:**

It is possible that 3-4 students may be able to complete the entire interview. If this happens, the SP is prepared to replay the scenario but to have a different viewpoint. You will need to tell the SP, outside of the room, this is what you want.

In the 2^nd^ scenario, the SP will be the same character but will be adamant about wanting everything done. The burden will be on the student not to take that at face value and to really explore the patient’s beliefs and provide informed consent.

**Other options**:

1. Ask the SP to have different beliefs-they would want CPR but never to be on a ventilator (a common conflicted desire that doctors face).

You might pose the following questions to the students for discussion.

What do you do when you have strong feelings about the choice the patient makes?

What do you do when the patient readily agrees to what you think they should do, or it seems too easy?

**TOTAL TIME: ~130 minutes**

Reminders About Time outs and Feedback

1. Time outs can be called by interviewer, patient, or faculty.
2. Person who calls time-out identifies reason for doing so (even if that reason is, time is up)
3. If student calls time out, ask, “How are things going? What made you stop? What are you stuck on?” etc.
4. Discuss the current challenge:

-student reflects on his or her interviewing

-observers share insight based on what they have observed or written down

-group brainstorms techniques to overcome the challenge the student is stuck on

1. Depending on how much time has elapsed, give the student the choice of rewinding to just before the point of frustration or to try and recover from the stuck point or have another student sub-in
2. When directing feedback, remember 1^st^ to ask the student what is going well before focusing on the negative. Once the interviewer has had a chance to debrief, ask if it is okay to gather observations from the group, and then yourself. Encourage the other students to offer their observations as well.

**RELEVANT Behaviors for Advance Care Planning Discussions**

**I Preparation**

Ensure appropriate setting and check that relevant people can all attend

**II Introduction**

1. Explain why you have convened this meeting now
2. Explain that its purpose is to respect patients’ wishes.
3. Reassure that death is not believed to be imminent (if true) – and avoid false reassurance.
4. Check how the patient feels about a discussion of advance care planning
5. Establish trust

Consider use of **PEARLS** for relationship building.

**P**-Partnership: (joining with patient) “Let’s tackle this problem together.”

**E-** Empathy (imagining/appreciating a patient’s unique circumstance and communicating your understanding to them back in a caring fashion)

**A-**Apology: (showing concern for someone’s difficulty with or without taking responsibility for it) “I’m sorry you’ve had such a difficult time with your illness.”

**R-**Respect: (showing appreciation for patient’s personal choices/behaviors, even if you disagree) “I appreciate that you’ve thought through this carefully and feel strongly about it.”

**L-**Legitimating: (normalizing and validating feelings and choices) “Anyone who went through that experience would feel equally frustrated.”

**S-**Support: (Offering encouragement, ongoing relationship)

**III Information**

1. Ask about patient’s understanding of the illness and prognosis
2. Provide additional information, using understandable terms
3. Clarify shared understanding of illness and prognosis.
4. Ask for patient’s understanding of likely further curative or palliative treatments
5. Provide additional information regarding likely treatments
6. Ask if the patient has ever documented their choices about life-sustaining treatment and ask what it says.

**IV Elicit Preferences**

1. Explore patients’ knowledge of other people’s end of life care, and their feelings about it.
2. Ask patients “What makes life worth living,” or “What should be the goals of treatment at the end of life?”
3. Elicit patients’ specific preferences, and ask “why?”
4. Identify what life states patients find unacceptable (e.g. Persistent Vegetative State) and what risks they are prepared to take to avoid these states.
5. Discuss probabilities and ask how patients would manage uncertainty.
6. If patients say they would not want “any” treatment, ask specifically about artificial nutrition and hydration.
7. Tell what you **WILL** do to meet patients’ goals and emphasize that you will remain involved regardless of what goals patients choose.

**V Proxies**

1. Identify who will be proxy (one person or a group?).
2. Explore how much communication there has been with the proxy.
3. Stress the need for good communication with the proxy.
4. Ask how much leeway proxy should have in decision-making.

**VI Documentation**

1. Make chart note of key discussion points.
2. Arrange for drafting and signing living will.

**VII Relationship**

1. Watch for emotional signals and respond verbally and non-verbally.

Consider using ***NURS*** mnemonic for emotion handling (Smith, RS).

**N-**Name the emotion.

**U-**Understand the emotion. “Help me understand more about why you’re feeling…” or “Given what happened, I can understand…”

**R-**Respect the emotion.

**S-**Support the patient. Problem solving or affirmation that you are willing to help.

1. Reassure patients that uncertainty and confusion is common and that they can have as much time to reflect as they need
2. Summarize the conversation (include unresolved issues) and check for shared understanding.

**SECTION III: SP Feedback and Wrap-up**

**FACULTY INSTRUCTIONS:**

**After the final student has interviewed, the SP will step out of the room to jot down final notes for feedback.**

**Do feedback process with the final student.**

**Bring SP back in the room and allow them to give feedback to students for ~8-10 minutes.**

**Ask students for any final thoughts or questions. Or ask what surprised them about the exercise or what they found particularly helpful. You may want to share also or offer a word of appreciation.**

**If you finish early, that is okay.**

**TOTAL TIME: 15 minutes**

Discussing Life-Sustaining Treatment, Pediatrics

Faculty Guide

**Session Objectives:**

1. Demonstrate ability to establish trust and rapport *prior* to and *during* conversations about life-sustaining treatment
2. Demonstrate a focus on patient’s or patient’s surrogate’s values and goals (rather than on treatment options) for advance care planning through a conversation with a patient about preferences for life-sustaining treatment
3. Use appropriate elements of informed consent in a conversation about life-sustaining treatment
4. Probe patients for clear understanding of meaning of preferences and goals

**Outline and Time Guideline for Session:**

| Activity | Page | Time Guideline |
| --- | --- | --- |
| I. Introductions and session set-up | 3 | 15 minutes |
| II. SP interview for practice | 4-9 | 100-110 minutes |
| III. SP feedback, wrap-up, debrief  **SECTION I: Introductions and Session Set-up** | 10 | 15 minutes |

**FACULTY INSTRUCTIONS:**

1. **Upon entering the room, please do not move the 2 chairs by the desk.** The interviewer should sit in the chair that faces the back door. The SP should sit in the chair that faces the students. It helps the students relax if they don’t have to face their peers during the interview. It also ensures the right camera angle and SP flow of traffic.
2. **Introduction:** Students will know each other and may have been in a small group together earlier in the week. Consider starting with an introduction of yourself and a very quick ice breaker (*i.e., have students introduce the person to their right and state what type of residency they are entering*). You might also consider sharing a brief story about yourself and an experience you had with discussions about life-sustaining treatment during residency or more recently that had a lasting impact on you.

Even in their 4^th^ year, students may have strong negative or positive feelings or anxiety about standardized patient activities. Probe for those and allow them to vent for a minute if needed.

1. **Session Set-up:**
   1. **Give outline of schedule**

It is expected that each student will take a turn at interviewing. There are 105 minutes available for interviewing, which gives each student about 17 minutes for both interviewing and feedback.

- 1. **Remind students of basic ground rules for these types of groups.** These should be very familiar to them, but it never hurts to explicitly state them at the beginning of a group.
- Respect different points of view and confidentiality of peers
- Be an active listener
- Feedback should be behaviorally based
- The person in the “hot-seat”, the SP, or the faculty member may call “time-out” at any time. The rest of the observers may not.

**TOTAL TIME: 15 minutes**

**SECTION II: SP Interview for Practice**

**FACULTY INSTRUCTIONS:**

1. **Ask students if they have any questions about the scenario.** They should have read the handout just prior to the session. (See below) Remind them that their end goal is to discuss preferences for CPR, but that may take several interviewers and the patient’s father may not come to a firm decision.
2. **Review the communication skills guide.** Observers should refer to this during the interview to serve as a guide for feedback. Encourage students to write down observations to guide feedback to their colleagues. Does the interviewer follow any of the steps listed? If so, ask for specific instances and perception of the impact on the patient. Does the interviewer respond to emotions, display empathy, and build trust? Ask observers to watch for specific examples.
3. **Ask students if they have questions left from the morning lecture.** Or ask if there are particular parts of the discussion they’d like to work on before. ***You may want to take some time to share a bit of your expertise on this topic as it relates to pediatric patients. In the lecture, the basic communication skills discussed apply regardless of age, but some of the other content is adult focused. It would help students if you briefly described how this encounter is different with pediatric patients/families and special considerations you keep in mind in your own practice.***
4. **Ask for one student to volunteer as the first physician to interview.** *Ask the student if there is any particular skill they’d like to work on and receive feedback about. Or ask them what they think a successful encounter would look like for their brief section of the conversation in specific terms. If they say no, that is okay too for this exercise.*
5. **Bring in SP and Introduce patient.** Mr. Ward is the father of 9 year old Kelly, who was admitted with an intracranial hemorrhage.
6. **Student interviews.** Use your judgment regarding time. One suggestion is to give 8-10 minutes for interview and approximately 8 minutes for feedback/debrief. Be cognizant of time constraints in an effort to give every student a chance to interview. This may require judiciously calling time-outs.
7. **Call time out.** See below. If student does not call time out before 8-10 minutes have elapsed, call time out. If the student calls time out before time is up, ask why, trouble-shoot, and give student option to rewind to just before point of frustration or they can try to recover from the stuck point.
8. **Have SP step out of room during time out between students.** This gives them a chance to jot notes for feedback. Upon resumption, this also gives you a chance to ask SP for any modifications outside of room just prior to next interviewer. When the next student is ready, you should invite the SP back in. If a student times out early, and is going to replay a part, the SP does not need to step out.
9. **Feedback**-see below for tips
10. **Ask for next volunteer**-Give them the option to rewind and replay or to pick up where the student left off.  *Ask if there is any particular skill they’d like to receive feedback about.* The SP will assume all interviewers are 1 doctor and there will not be a need for students to introduce themselves each time or restart relationship-building.
11. **Repeat steps 6 through 9** until all students have interviewed or time is up. DO NOT have each student restart the scenario from the beginning.
12. **If the scenario is resolved after 2-3 students, you may privately ask the SP if they could restart the scenario with a different attitude. The details will be the same, but the SP will be able to vary their opinions and responses.**

SECTION II: SP Interview for Practice

Reminders About Time outs and Feedback

- 1. Time outs can be called by interviewer, patient, or faculty.
  2. Person who calls time-out identifies reason for doing so (even if that reason is, time is up)
  3. If student calls time out, ask, “How are things going? What made you stop? What are you stuck on?” etc.
  4. Discuss the current challenge:

-student reflects on his or her interviewing

-observers share insight based on what they have observed or written down

-group brainstorms techniques to overcome the challenge the student is stuck on

- 1. Depending on how much time has elapsed, give the student the choice of rewinding to just before the point of frustration or to try and recover from the stuck point or have another student sub-in
  2. When directing feedback, remember 1^st^ to ask the student what is going well before focusing on the negative. Once the interviewer has had a chance to debrief, ask if it is okay to gather observations from the group, and then yourself. Encourage the other students in the group to give feedback as well.

**RELEVANT Behaviors for Advance Care Planning Discussions**

**I Preparation**

**1.** Ensure appropriate setting and check that relevant people can all attend

**II Introduction**

1. Explain why you have convened this meeting now
2. Explain that its purpose is to respect patient’s wishes.
3. Reassure that death is not believed to be imminent (if true) – and avoid false reassurance.
4. Check how the patient feels about a discussion of advance care planning
5. Establish trust

#1-4 can do this, in addition, consider use of PEARLS for relationship building.

**P**-Partnership: (joining with patient) “Let’s tackle this problem together.”

**E-** Empathy (imagining/appreciating a patient’s unique circumstance and communicating your understanding to them back in a caring fashion)

**A-**Apology: (showing concern for someone’s difficulty with or without taking responsibility for it) “I’m sorry you’ve had such a difficult time with your illness.”

**R-**Respect: (showing appreciation for patient’s personal choices/behaviors, even if you disagree) “I appreciate that you’ve thought through this carefully and feel strongly about it.”

**L-**Legitimating: (normalizing and validating feelings and choices) “Anyone who went through that experience would feel equally frustrated.”

**S-**Support: (Offering encouragement, ongoing relationship)

**III Information**

1. Ask about patient’s understanding of the illness and prognosis
2. Provide additional information, using understandable terms
3. Clarify shared understanding of illness and prognosis.
4. Ask for patient’s understanding of likely further curative or palliative treatments
5. Provide additional information regarding likely treatments
6. Ask if the patient has ever documented their choices about life-sustaining treatment and ask what it says.

**IV Elicit Preferences**

1. Explore patients’ knowledge of other people’s end of life care, and their feelings about it.
2. Ask patients “What makes life worth living,” or “what should be the goals of treatment at the end of life?”
3. Elicit patients’ specific preferences, and ask “why?”
4. Identify what life states patients find unacceptable (e.g. Persistent Vegetative State) and what risks they are prepared to take to avoid these states.
5. Discuss probabilities and ask how patients would manage uncertainty.
6. If patients say they would not want “any” treatment, ask specifically about artificial nutrition and hydration.
7. Tell what you WILL do to meet patients’ goals and emphasize that you will remain involved regardless of what goals patients choose.

**V Proxies**

1. Identify who will be proxy (one person or a group?).
2. Explore how much communication there has been with the proxy.
3. Stress the need for good communication with the proxy.
4. Ask how much leeway proxy should have in decision-making.

**VI Documentation**

1. Make chart note of key discussion points.
2. Arrange for drafting and signing living will.

**VII Relationship**

1. Watch for emotional signals and respond verbally and non-verbally.

Consider using ***NURS*** mnemonic for emotion handling (Smith, RS).

**N-**Name the emotion.

**U-**Understand the emotion. “Help me understand more about why you’re feeling…” or “Given what happened, I can understand…”

**R-**Respect the emotion.

**S-**Support the patient. Problem solving or affirmation that you are willing to help.

1. Reassure patients that uncertainty and confusion is common and that they can have as much time to reflect as they need
2. Summarize the conversation (include unresolved issues) and check for shared understanding.

**SECTION III: SP Feedback and Wrap-up**

**FACULTY INSTRUCTIONS:**

**After the final student has interviewed, the SP will step out of the room to jot down final notes for feedback.**

**Do feedback process with the final student.**

**Bring SP back in the room and allow them to give feedback to students for ~8-10 minutes.**

**Ask students for any final thoughts or questions. Or ask what surprised them about the exercise or what they found particularly helpful. You may want to share also or offer a word of appreciation.**

**TOTAL TIME: 15 minutes**
