# Supplementary material for: End-of-Life Care: A Multimodal and Comprehensive Curriculum for Graduating Medical Students Utilizing Experiential Learning Opportunities
Source: MedEdPORTAL. 2021 Apr 27;17:11149. doi: 10.15766/mep_2374-8265.11149 (PMC8076371; doi:10.15766/mep_2374-8265.11149)
Supplement: Supplementary file 1 — End-of-Life 1 Faculty Guide.docxEnd-of-Life 1 Student Handouts.docEnd-of-Life 1 Standardized Patient Materials.docxEnd-of-Life 2 PowerPoint Presentation.pptEnd-of-Life 2 Faculty Guide.docxEnd-of-Life 2 Simulation Materials.docxEnd-of-Life 2 Simulation Case Faculty Guide.docxEnd-of-Life 2 Standardized Patient Materials.docxEnd-of-Life Assessment.docx [file mep_2374-8265.11149-s001.zip › C. End-of-Life 1 Standardized Patient Materials.docx]

# EOL I Adult Case Life-Sustaining Treatment: Ben/Benita Ward

# CASE SUMMARY

**OVERVIEW**

**Objective:**

To enhance fourth year medical students’ skills in discussing goals and preferences for life-sustaining treatment with a hospitalized patient.

**Competencies Assessed:**

1. Patient education and counseling skills

2. Negotiation and shared decision-making skills

3. Relationship development skills

4. Conflict resolution skills

5. Global satisfaction

**Logistics**:

**Personnel**- SP, preferably age >50, hospital gown, in chair

**Station Materials:**

1. Instructions for the examinee

2. Fact sheet for the examinee

3. SP instructions

4. Nasal cannula for oxygen

5. SP to be dressed in hospital gown and pants/socks or pajama bottoms/socks

**Room arrangement:**

1. Door sign (or some other way to direct students and faculty to correct room) indicating faculty and students

2. Outpatient exam table/hospital bed

3. Chair next to the exam table

4. Chair/stool for doctor

5. Chairs for small group of students/faculty

**STANDARDIZED PATIENT INSTRUCTIONS**

**Ben/Benita Ward**

Learners: End-of-fourth-year medical students, residency match happened recently

Students are in groups of four-six, with a faculty preceptor

- SP enters and leaves the room for each student; student is considered to be the same clinician each time
- Approximately 10 min of encounter/student, preceptor controls the timing, approximately 10 min feedback from preceptors to students and prep for next encounter
- SP makes notes for feedback between encounters and gives feedback on interpersonal skills at the end (20 minutes for feedback at the end)
- The preceptor gets the SP for re-entering. At that time, ask the preceptor what is happening with the encounter

**The Scenario**

Four days ago, you became more short of breath than usual and started to cough up more phlegm. Yesterday, when you started to feel short of breath when just sitting down, you went to the emergency department. You were told you are having a flare up of your emphysema. You were started on medication and you are feeling better. You have been in the hospital since yesterday. You met the resident yesterday. They are now being asked to follow up with you regarding advance directives.

This whole episode has happened to you before. In fact, this is the second time in nine months you have been in the hospital for the same problem and the fifth time overall since you were first diagnosed with emphysema 10 years ago. Every time this happens, you get some medicine and feel better within a week, so you figure it is not that serious and that it will be treatable every time. You smoked a lot, two-three packs per day for almost 45 years but quit about two years ago.

You know your breathing has not been good, and every year it seems to get worse. You used to play sports with your kids, but in the past year you noticed you have to stop and rest when you get to the top of a flight of steps. You are worried about this happening, but no one has ever told you your emphysema is “severe”, and you figure that there must be other medicines you can take. At the very least you think you could live another 10 years.

Recently your primary care doctor mentioned the emphysema was “getting bad” and that at the next visit you should talk about your “living will”, but you’re not sure why your doctor wants to know about your finances. The resident in the hospital told you they’d “have you out of here in no time”, the nurse told you “take your medicine and everything will be fine” and the pulmonary doctor told you that you might need oxygen all the time from now on. You are very confused and don’t understand why everyone is saying something different.

You want to believe you are going to be fine and live several more years. You tend to believe the people who reassure you and think the pulmonary doctor must not know what he’s talking about. He’s only met you once, so how could he possibly know anything about you? He used a lot of big words and you didn’t understand half of what he said anyway, but you were embarrassed to admit that to him.

You really want to live to see your grandkids grow up and graduate from high school. You worked a lot when your kids were younger, so now you are really enjoying the chance to spend time with their kids.

***Past Medical History***: Emphysema (COPD) for which you have used inhalers for 10 years, and high blood pressure for which you take hydrochlorothiazide (HCTZ) or “water pills.”

***Family History:*** The doctors always ask you about “diseases that run in your family” so you tell them about your parents who both died from heart attacks. If you are asked about ‘bad’ deaths, your older brother was in a car accident about three years ago and had severe injuries. He was hooked up to life support (on a ventilator) for three weeks before he died. It was very hard for you to see your brother like that and you were very concerned that he was in pain and suffering. After that you are not sure you would ever want to be on a ventilator but at the same time you want to live for a long time.

***Social History:*** You are 65 years old. Married for 30 years, live with your spouse in Arbutus (suburb). You have two children and two grandchildren and work as a delivery person for a linen service that supplies local hospitals and nursing homes. You’ve worked there for 25 years. When your children were younger and living at home, you worked two jobs to support your family, but now are just working for the linen company. You completed the 11^th^ grade. You quit smoking two years ago. You do not drink alcohol. You spend a lot of time with your grandchildren and often babysit them on the weekends when their parents are working. You were raised Methodist, but you don’t have particularly strong religious beliefs.

**The Medical Encounter**

**Challenges for student:**

You are very confused by all the different opinions you have been told, and if the student does not gain your trust, you will be skeptical of what they say. See below for details.

You are scared about what will happen to you because of your emphysema and that fright may translate into some anger if you think the doctors are “giving up” on you.

You only completed the 11^th^ grade and have a somewhat low health literacy level.

Opening statement: Depends on what student says

| ***Introducing topic, describing/defining advance directives, rationale*** | What you do is … |
| --- | --- |
| If, without establishing trust or explaining, the student starts by saying they want to talk to you about your “code status”, “CPR”, advance directives, or whether you want to be resuscitated … | This will make you confused and scared (which translates into being mildly angry) because it will make you think you must be dying. Abruptly say “Yes, I want everything done.” This will shut you down a bit and the student will have to recognize your emotion and work to regain trust. |
| If the student says above but then qualifies by saying “This is something we talk to everyone about in the hospital.”  A positive behavior would be for the student to explain why now, and that they don’t believe you are imminently dying, and ask if it is okay to discuss something with you.  Other positive behaviors: explains that it is a patient’s right to make decisions about their own care OR they are talking to you about this because they want to be sure the medical team knows what the patient wants and to prevent care the patient does not want. | Ask “What for?” |
| If the student starts the conversation by being falsely reassuring, “I think you’re going to be fine” or “You’re not going to die any time soon” | Latch on to that and press the student about it. “Oh good, so you think I’m going to be fine. That’s not what that lung doctor said, but I don’t think he really knows anything.” |
| Positive behaviors that would gain your trust:  a) Relationship building: empathy, legitimizing, partnering, naming, etc.  b) Asking you your understanding of the situation  c) Acknowledging that there is a lot going on while you are acutely ill, OR you don’t know each other well and makes an attempt to get to know what you do outside the hospital.  d) Demonstrates respect (I’m impressed by…”)  e) Humility (“I appreciate you sharing this with me…”) | “I know I have lung problems, but I can still do a lot.”  “I think my doctor thinks I’m fine.” “Several people here told me I would be fine.”  “I thought I would be okay, but people here keep telling me different things. I don’t know who to believe.”  “There must be some medicine that can make me feel better.”  “I think I could live for a long time if I take care of myself.” |
| If student contradicts or puts down other health care providers …  A positive behavior could be:  “I hope you are right, but have you thought about what might happen if things don’t go as you wished” or “I wish too that you will live a long time. If it turns out that your lungs are too bad and you cannot, are there shorter-term goals that are important to you?” | Depending, you will join in or grow more confused/angry. If the student says something like “Sounds like Dr. X left you feeling very hopeful. I’m sure they really care about you…” you will not get angry if they later contradict Dr X. |
| At some point, you and the student should come to a shared understanding of illness and prognosis.  (Based on some prognostic models, risk of death from any cause 10-25% in next 3 years, chance of further recurrences 100%) | If student explains it in clear terms and asks you for your opinion or thoughts about it, you will accept what they have to say. “Thanks for being honest with me, doc” or “I guess everyone has their time.” Or similar statement |
| The student should set the stage and explain to you they want to talk about “advance directives”, a “living will”, preferences for care, etc.  If the student introduces a term but doesn’t explain what it means, answer with a confusing statement…  If they provide a definition, if you feel it is clear, say okay or ask a clarifying question. | “I have a will; I’m leaving everything to my wife/husband”  “Yeah, that’s the thing where you set up your burial ahead of time. I haven’t done that.” |
| If the student asks you if you have a living will, advance directive, or health care proxy, if they haven’t explained the term, use one of the confused statements above.  If they have explained the terms, and ask if you have one of the above… | “No, no one ever talked to me about it” OR “My doctor mentioned last visit we should talk about it next time.” |
| If student launches directly into asking you if you would want CPR, intubation, etc.… | “Of course, I want to live” and shut down a bit unless student explores with you the options or your feelings about it. |
| If the student asks you if you have known someone who had to go on a ventilator, get CPR, or seen someone who had a “bad death” | Say “Yes, that happened to my brother.” Do NOT disclose this unless they ask. AND do not discuss your feelings about that scenario unless they ask. |
| If they ask you about someone who had a “good death”… | “My grandmother died in her sleep at home.” |

| ***Assessing goals*** |  |
| --- | --- |
| “What is your life outside the hospital like?”  “What are the most meaningful or enjoyable parts of your life?”  “What makes life worth living for you?”  “What do you consider your quality of life to be like now?”  “What are you most important hopes?”  “What are your biggest fears?”  “If you were to die sooner rather than later, what would be left undone?” | Describe life being very involved with grandkids, working although that’s getting much harder. You think your quality of life is “pretty good” although a bit frustrated you can’t physically do everything you used to.  Most important thing is seeing grandkids grow up, graduate from school, etc.  Biggest fear is being a “burden” and “suffering” |

| “Are there any circumstances that would make life not worth living for you?” | Wouldn’t want to live like a “vegetable” |
| --- | --- |
| You will use some general phrases, and it is important that student ask for clarification about what you mean.  Positive questions:  “Why do you feel that way?” “Tell me what that means to you.” “What is it about that quality of life that is intolerable to you?” |  |
| “Vegetable” | You don’t want your life prolonged if there is little or no chance of having a decent quality of life. This means the ability to talk and carry on a conversation, to interact with your family, to be part of your grandkids’ lives, which means not being in a nursing home and not being “hooked up to machines” |
| “Burden” | You don’t want your spouse or children to have to provide much in the way of physical care for you (i.e. bathing you, feeding you) and you do not want to place any financial strain on them if they had to pay for a nursing home or hire people to care for you. |
| “Suffering” | Feeling like you can’t catch your breath, having a lot of pain, being “locked up” in a nursing home or on machines, knowing your grandkids were growing up but you couldn’t see them” |
| “Hooked up to machines” | Feeding tubes, ventilators |

The student should be able to transition from your goals to a discussion about what you might want if you needed resuscitation. They may do this by posing scenarios to you, or they may ask more generic questions. The goal of the conversation is not necessarily to come to an agreement. After they pose choices to you, it’s okay to say you need some more time to think about it or discuss it with your spouse, if that’s how you feel.

However, it is important that the student do two things:

1) Explain cardiopulmonary resuscitation (CPR), intubation, etc. to you in terms of informed consent (risks, benefits, alternatives), and

2) Before the end of the conversation, at least pin you down on who your health care proxy should be.

| ***Discuss scenarios, explain risks and benefits of proposed interventions*** |  |
| --- | --- |
| “Dire consequences scenario”  Severe brain damage, massive heart attack, widely metastatic cancer (there may be others),chance of recovering to your “quality of life” <1 or 2%, “almost no chance” | You would not want CPR, ventilation, tube feeding in that situation |
| “Reversible scenario”  Needed minor surgery, chance of recovering to your quality of life “excellent” or very good” or >75% | You would want everything in that scenario |
| “Uncertain scenario”  Bad pneumonia, stroke, pancreatitis, heart failure, chance of recovering to your “quality of life” is “hard to predict”, could live but higher chance of brain damage, dependence and debility | You’re not sure. You don’t divulge this, but part of the reason you are not sure is because you worry that if you did not choose intubation, etc., the doctors would “give up” and let you suffer |
| If the student assures you that even if you didn’t choose life sustaining measures the team would work aggressively to make sure you are comfortable OR if they ask what makes you unsure or about concerns you might have…  If they do not reassure your fears or probe… | You will feel relieved and say that you’re glad to have the discussion and will talk more with your spouse about it.  You will try to hasten the end of the conversation. |

| ***Forms, health care proxy*** |  |
| --- | --- |
| If asked, who should make decisions about your healthcare for you if you couldn’t speak for yourself… | Your spouse and then your daughter |
| If asked if you’ve ever discussed this with them before… | No |
| If asked whether it was more important for them to follow your wishes strictly, or make slightly different decisions in your care if they felt it was right… | You trust your family to what is best |
| If they offer you a form to look at or tell you they will get one for you… | Be grateful, but also ask if you can change your mind later about this. |

Again, at the end of the conversation, it is not absolutely necessary for you to have made a decision about your life sustaining treatment.

The most important thing is that at the end of the conversation, you felt heard, things were explained in clear terms using informed consent principles, the student made an effort to understand your thoughts, and they displayed empathic behaviors and worked to build trust.

The bottom line is you previously were afraid your emphysema might be bad but had not had an honest conversation about it.

Right now, you feel like you have a lot to live for and would be willing to try some aggressive treatment if there was chance of benefit.

**ALTERNATIVE SCENARIOS**

There will be four-six students in the group. It is possible by the close of the third or fourth student, that the scenario may feel complete. In this case, the instructor may ask you to restart the encounter but with different opinions.

You will be the same patient with the same back story. However, now when asked about your preferences, you will say you want “everything done” no matter what is offered or how it is presented. You really believe that CPR works as well as it does on television. In this scenario, your brother will not have been on life support and you have no prior personal experience with a loved one on life support.

It will be up to the student to explore your reasons (same as above, want as much time as possible with your grandkids). You don’t want to be a “vegetable” or a “burden” but don’t understand that could possibly happen with CPR and intubation.

It will also be up to the student to really probe what your understanding of your illness is. They will need to present you with the facts above about prognosis. They should ask you if hearing those facts changes your mind. If they do, you could say, “I need to talk with my wife about it.”

It is possible that there will be details that do not get revealed in the first run through; you could also reveal some of those details, if congruent with this version of the patient.

Some **positive behaviors** on the student’s part might include:

- Giving you time to absorb and to speak (looking at you, not rushing to fill any silence.)
- Showing empathy
- Compassionate listening (nodding, allowing you to speak, and maintaining warm eye contact)
- Recognizing and responding to your emotional signals (“Is talking about this difficult for you?” “Making these decisions is not easy. I wonder if it feels overwhelming for you?”)
- Building trust by acknowledging conversation is awkward, expressing partnership with you, making effort to get to know you as a person
- Legitimizes your feelings (“It’s difficult for many people to make these decisions” “it’s understandable that talking about this may make you worry, feel sad, angry, etc.”)
- Offers support (“I will make sure we have time to talk about this again if you like” “I can help you talk to your primary care doctor about this”)
- Explores statements you make or emotions you display (“Tell me more about…” “What does ____ mean to you?”)
- Expressing terms in clear simple language. Checking to see if you understand (teach back, probing question)

Some **negative behaviors** that would make you scared and thus mildly angry or confused, might include:

- Rushing
- Doing most of the talking without exploring your feelings or beliefs
- Not recognizing your emotional cues or displaying empathy
- Using confusing medical terms
- Making you feel like it is not okay to have hope

**SP Instructions on Discussing Life-Sustaining Treatment**

**Flow**

Initially:

Confused as to why discussing now

Confused by conflicting messages

Confusion makes you mildly angry

If student demonstrates repeated negative behaviors, you will either become mildly angry or try to shut down the conversation.

You are the same character throughout all encounters, but you should give some different information to each student and vary your reaction slightly with each student (e.g., sarcasm vs. threats). Save some of your material for subsequent students.

If student demonstrates consistent, positive behaviors, you might disclose some of your fears—e.g., “I don’t want to die, but I don’t want to be a burden.”

RELIEF THAT YOU FEEL

SUPPORTED, ONLY SLIGHTLY

VISIBLY SAD, BUT FEELING SAD,

GUILTY, TRYING TO STAY STRONG IRRITATION / FRUSTRATION/SHUTDOWN

**Emotional modulation: Moderate** 2-3/10 (0= flat, 2-3=normal; 10=intense):

You are confused by conflicting messages, timing of conversation, which makes you either shut down or mildly angry, but if the student earns your trust, your affect is normal.

**References**

Tulsky JA. “Beyond advance directives: Importance of communication skills at the end of life.” JAMA 2005;294:359-65.

Roter DL, et al. “Experts practice what they preach.” Arch Intern Med 2000;160:3477-85.

Quill TE. “Initiating end-of-life discussions with seriously ill patients.” JAMA 2000;284: 2502-7.

Tulsky JA, et al. “Opening the black box: How do physicians communicate about advance directives?” Ann Intern Med 1998;129:441-9.

Tulsky JA, et al. “How do medical residents discuss resuscitation with patients?” J Gen Intern Med 1995;10:436-42.

# EOL I Pediatrics Case Life-Sustaining Treatment: Kelly Ward

# (parent: Sam Ward)

# CASE SUMMARY

**OVERVIEW**

**Objective:**

To enhance fourth year medical students’ skills in discussing goals and preferences for life-sustaining treatment with the parent of a terminally ill, hospitalized child. (ACGME competency 4: interpersonal/ communication skills)

**Competencies Assessed:**

1. Patient education and counseling skills

2. Negotiation and shared decision-making skills

3. Relationship development skills

4. Conflict resolution skills

5. Global satisfaction

**Logistics**:

**Personnel**- 1 SP, to portray parent of a 9-year-old child

Group of four-six students with a preceptor

**Station Materials:**

1. Instructions for the examinee

2. Fact sheet for the examinee

3. SP instructions

**Room arrangement:**

1. Door sign with preceptor and student names

2. Chair next to the exam table

3. Chair/stool for doctor

**STANDARDIZED PATIENT INSTRUCTIONS**

**Sam (Kelly) Ward**

**The Scenario**

Your daughter, Kelly, is a 9-year-old girl who was diagnosed with a brain tumor eight months ago. Soon after the tumor was diagnosed, the doctors tried to remove it all through surgery. Unfortunately, the entire tumor could not be removed. She had a shunt placed in her brain in order to drain fluid from her ventricles to treat “water on the brain” (hydrocephalus) that is a side effect of the residual tumor that could not be removed. This was not a treatment for the tumor, just a measure to try to prevent this complication of the residual tumor.

Because the entire tumor could not be removed, Kelly has been undergoing chemotherapy and radiation, for almost the entire last seven months. This requires her to go to the hospital several times per week for treatment. Kelly seems tired a lot of the time but has managed to keep going to school despite all this. She has been hospitalized twice since the initial surgery, both times for infections (pneumonia).

Two days ago, you were at home with Kelly playing on the Wii. Very suddenly, Kelly fell and had trouble standing. Her speech was garbled, and she was not making any sense. You immediately called 911 (emergency services) and an ambulance arrived within 15 minutes.

When she got to the hospital, the doctors ran tests on Kelly and determined that she had a large amount of bleeding in her brain around the area of the tumor. This bleeding has caused pressure in the brain and is causing her brain to swell, which, you understand, means that there may not be enough room in her skull for her brain, causing brain damage. The neurosurgeons have said that there is no surgery that will relieve the pressure without doing further damage. You have been told that her chances of recovering are “slim”.

Currently, Kelly is sleeping most of the time, but you can see that she tries to open her eyes when you call her name or squeeze her hand. She is on a breathing machine (ventilator). You have been at the hospital almost constantly since this happened two days ago.

Kelly’s usual neuro-oncologist is out of town. The pediatric intensive care unit (PICU) doctors, including one of the residents, said they want to talk with you about what Kelly’s prognosis is and what kinds of treatment they should provide her with. You wish her regular doctor was in town, but also really want information, so you are satisfied that the PICU doctors want to take time to meet with you. You have met with the PICU doctors a couple times, but today new residents rotated onto the service. You have not met the residents who want to meet with you before.

You are very scared by this event. You have known and know now that things are not good for Kelly given how aggressive the tumor is that she has, but you are really hoping she will pull through this. She was doing so well, all things considered, before this, despite what the doctors had said about how aggressive this type of tumor is. Your wife died in a car accident two years ago, and Kelly is your only child. You feel desperate to hold on to Kelly and keep her alive both because you lost your wife and because you promised your wife, Krista, that you would take care of Kelly. You really want to be a good parent for Kelly. It also is very important to you that Kelly live until your sister’s wedding, which is in six months. Kelly is going to be the flower girl and has been very excited about this.

However, seeing Kelly on the ventilator is very upsetting to you. As much as you want Kelly to pull through, you know that Kelly is happiest when she can go to school and see her friends and play with her dog. As hard as it is for you to admit it, if Kelly was not able to do those things ever again, you know that it would not be right to put her through a lot of painful treatments just to keep her with you. You wonder if being on the ventilator is painful.

*As upsetting as all of this is, you try not to let other people see how upset you are. When you talk with the doctors, you often feel like you are in “shock” (numb, like you can’t believe what they are saying). You think you must “stay strong" and rather than breaking down, if you start to feel emotional during the conversation with the doctors you may show some frustration, but it is very unusual for you to cry in front of Kelly’s doctors. This has been true ever since her initial diagnosis.*

You are not at all ready to take Kelly off life support. You really want to see if she starts to improve in the next couple of days. The doctors are going to ask you if you would want Kelly to have “CPR” (or chest compressions, or resuscitation, etc.) if she were to deteriorate and her heart stopped beating. Initially you are not sure what to say, but as the conversation goes on, depending on how it is handled, you start to wonder if people pounding on the chest of your little girl is what she would want or what your wife would have wanted, especially if there was a good chance it would not help her get back to playing with her friends. You don’t know much about CPR, except what you have seen on television (it either works like a miracle and the person gets back to normal or the person dies).

Even though she had a significant medical history, life sustaining treatment was not discussed with you or her previously. Clinicians can be reluctant to bring up the topic unless it needs to be discussed.

***About Kelly***: Kelly had a very healthy childhood until eight months ago when she had a seizure for the first time. When she admitted to the hospital, they found out she had a brain tumor. She had surgery to remove it, but it didn’t get the entire tumor out. She has been getting regular radiation and chemotherapy since. For the most part, Kelly has been a real trooper. She is a very loving child and is often more concerned about the people around her than herself. She complains sometimes about being sick, but as long as she can keep going to school, she seems okay. Her teachers and classmates have been very supportive. The last time Kelly was in the hospital for pneumonia (two months ago), she told you that she was tired of hospitals and doctors and didn’t want to do it anymore. You didn’t ask her what she meant; you were too scared. Since then, she hasn’t said anything else like that. You have never told Kelly that she might die; you are too worried that it might cause her to be depressed and give up.

Kelly was very close to her mother and initially struggled a lot after her death. Since then, the two of you have grown very close. Kelly is also close to her grandparents on both sides.

Kelly has some very close friends at school and spends a lot of time with them. She loves all animals, but especially her dog, Biscuits (a four-year old golden retriever). She used to like to play soccer but hasn’t since she got sick. She also loves pop music. She collects American Girl dolls. When your sister asked Kelly to be the flower girl in her wedding, she was ecstatic and has talked about it regularly since.

***About You:*** You work as an IT (informational technology) analyst for Constellation Energy (local utility supplier). You make a comfortable salary. Your job has been very supportive during Kelly’s illness, and fortunately, you can often work from home. You have a college degree, but sometimes find the complex aspects of Kelly’s treatment confusing.

You and your wife were married for 12 years before she died. You were married for five years before you ever had Kelly. Your wife was driving home from work one day and was killed instantly in a motor vehicle accident. You don’t have any personal experience with life support or decisions about life support through this.

Your parents are your main sources of support and have been since your wife’s death. You often consult with them when there are major decisions to be made about Kelly’s treatment.

You were raised Methodist, but don’t have any strong religious beliefs. You don’t have any desire to speak with a chaplain.

**The Medical Encounter**

**Challenges for student:**

Deep down, you know that Kelly will likely not survive this illness. You don’t want her to suffer but feel conflicted by your intense desire for her to live as long as she can. You feel very guilty that all of this happened, because you promised your wife you would take care of her.

You are not ready to make any firm decisions about withdrawing the ventilator or whether Kelly should have CPR. But, if the student acknowledges your distress, that you are good parent, you will be open to hearing the options, and start to consider saying that she should not have CPR if her heart stops beating.

Sam (Kelly) Ward:

Opening statement: Depends on what student says

| ***Opening the conversation, introducing topic, shared understanding of the current condition*** | SP Response |
| --- | --- |
| If the student starts by saying they want to talk to you about Kelly’s “code status,” “CPR,” advance directives, or whether she should be resuscitated without establishing trust | This will make you confused and scared (which translates into being frustrated) because it will make you think they are giving up on her. Abruptly say “Yes, I want everything done, I want her to live.” This will shut you down a bit and the student will have to recognize your emotion and work to regain trust. |
| If the student says the above but then qualifies by explaining that before that they want to know your understanding of the illness, or about Kelly, or how you are doing, or asks you to talk about something before launching into the discussion about CPR | You will be very tense, and be very slightly frustrated, because you worry they are giving up on Kelly, but you soften a bit and will answer the question. You are wary because these doctors don’t know Kelly. |
| A positive opening would include asking:  “What is your understanding of Kelly’s condition?”  “How are you doing?”  “Tell us something about Kelly.” | Recite what is written in the case description  *Answer to condition?*  “I’m ok, a little tired, and just very worried about my daughter.”  “Before all of this happened, she was going to school, doing sports, doing stuff with her friends.”  8 months ago, brain tumor, treatment, 2 months ago 2 times pneumonia, then 2 days ago…  (She loves her dog. I brought in a picture of him for her and put in by her bed.”) |
| It would be very helpful if the student acknowledged in some way that you are a good parent. (Acknowledge your devotion, attentiveness, advocacy, intentions to make the best decisions possible.) |  |
| Other helpful behaviors include:  Acknowledging that you know your child better than anyone on the medical team and assuring you that your values and wishes are going to be respected.  Giving you positive feedback for difficult  but loving decisions that you have made and your devotion to Kelly. |  |
| ***Assessing Goals*** |  |
| At some point student needs to bridge to discussion of goals  The student should transition into the discussion and explain to you they want to discuss goals of care. The language to introduce this may vary and positive examples could include “goals for Kelly’s care” or “”options for treatment if things don’t go well” or “options for treatment now that Kelly is very sick.” |  |
| If the student is vague about what you are about to discuss OR introduces a term but doesn’t explain what it means OR launches into questions about the topic (of CPR) but doesn’t warn you ahead of time🡪 answer with a question showing confusion. | Why are we talking about this?  What do you mean?  What really happens with CPR? |
| If the student asks you about preferences or goals of care, before you have a shared understanding of Kelly’s prognosis… | Say “I don’t know what you’re talking about. Her oncologist said she still has time left.” |
| If they provide a clear explanation of what they are asking you to discuss AND you feel like you understand Kelly’s prognosis and why they are asking you to discuss this now… | Respond nondefensively with openness to having the discussion BUT acknowledge that you are not ready to say Kelly may die. |
| A positive behavior might include asking you what your hopes for Kelly are OR what your fears for Kelly are. | Hopes: A miracle (but you know that may not be realistic), for Kelly to recover enough to stay with you a little longer, to be part of your sister’s wedding.  Fears: That she will suffer, feel pain or scared, be a “vegetable”, that you will make the “wrong” decision |
| Another positive behavior would be to try and understand what provides Kelly enjoyment and meaning in her life, and conversely, if there were things she could no longer do, that would detract from her quality of life.  “What are the most meaningful or enjoyable parts of her life?”  “What do you consider her quality of life to be like now?”, “What was it like just before this happened?” | She loves being with her friends and family, going to school, and playing with her animals.  When she has been really sick, and she can’t do those things she feels very sad.  Before this happened, her life was okay. She couldn’t do everything she used to do but did a lot of things to make her happy. Now it is so hard to see her hooked up to the ventilator. I don’t know what she is aware of, but I think she knows I’m here. |
| If the student asks you if you have known someone who had to go on a ventilator, get CPR, or seen someone who had a “bad death” or suffered at the end. | Answer to ventilator/CPR, “Only what I have seen on TV. It seemed pretty successful there.”  Answer to a bad death, “I don’t know of any.” “Any death is sad.” |
| If they ask you about someone who had a good death… | “My grandmother died in her sleep at home.” |
| “Are there any circumstances that you think would make life for her not worth living?” | Wouldn’t want to her live like a “vegetable” |
| ****You will use some general phrases, and it is important that students ask for clarification about what you mean.  Positive questions:  “Why do you feel that way?” “Tell me what that means to you.” “What is it about that quality of life that is intolerable to her?” If you use the following terms: |  |
| “Vegetable” | You don’t want her life prolonged if there is little or no chance of having a decent quality of life. This means the ability to talk and carry on a conversation, to interact with her family and friends |
| “Suffering” | Feeling pain or being scared or lonely. Being on the ventilator indefinitely. |
| “Hooked up to machines” | Feeding tubes, ventilators |

The student should be able to transition from your goals to a discussion about what you might want if Kelly needed CPR or resuscitation if her heart stopped.

The goal of the conversation is not necessarily to come to an agreement. If the student has displayed several positive behaviors, by the end of the conversation you will:

1. Not be ready to remove Kelly from the ventilator, you want to wait another day or two and see what happens
2. State you really want to think about everything that was discussed, and you want to speak with your parents about this
3. Possibly state that you do not want Kelly to have CPR (see below)

| ***Explain risks and benefits of proposed interventions*** |  |
| --- | --- |
| A positive behavior would be to explain what CPR is | You have some understanding from television. |
| If the student does not present any of the risks of CPR | You would want everything done |
| If the student discusses the fact that CPR is unlikely to restore Kelly’s quality of life | You acknowledge that you have concerns about putting her through that |
| A positive behavior would include:  When discussing what they think is best not to do (invasive death-prolonging interventions). Using such language to emphasize what is going to continue (e.g., pain control, symptom relief, family support). | You will feel relieved that Kelly would not be abandoned by them. |

Appearance: not shaven, wear a ‘wedding’ ring, rub your face with exhaustion

You can start standing or sitting. When the resident comes into the room, pace around.

Again, at the end of the conversation, it is not necessary or expected for you to have made a decision about life sustaining treatment.

The most important thing is that at the end of the conversation, you felt heard, things were explained in clear terms using informed consent principles, the student made an effort to understand your thoughts, and they displayed empathic behaviors and worked to build trust.

Some **positive behaviors** on the student’s part might include:

- Giving you time to absorb and to speak (looking at you, not rushing to fill any silence.)

- Showing empathy

- Compassionate listening (nodding, allowing you to speak, and maintaining warm eye contact)

- Recognizing and responding to your emotional signals (“Is talking about this difficult for you?” “Making these decisions is not easy, I wonder if it feels overwhelming for you?”

-Building trust by acknowledging conversation is awkward, expressing partnership with you, making effort to get to know you as a person

-Legitimizes your feelings (“It’s difficult to make these decisions” “It’s understandable that talking about this may make you worry, feel sad, angry, etc.”)

-Offers support (“I will make sure we have time to talk about this again if you like” “I can help you talk to your primary care doctor about this”)

-Explores statements you make or emotions you display (“Tell me more about…” “What does ____ mean to you?”)

-Expressing terms in clear simple language. Checking to see if you understand (teach back, probing question)

Some **negative behaviors** that would make you scared and thus mildly angry or confused, might include:

-Rushing

-Doing most of the talking without exploring your feelings or beliefs

-Not recognizing your emotional cues or displaying empathy

-Using confusing medical terms

-Making you feel like it is not okay to have hope

**SP Instructions on Discussing Life-Sustaining Treatment**

Initially: “IN SHOCK”

(numb, having a hard time believing what is happening, overwhelmed by situation)

- - - - - - - - - - -- - - - - - - - - - - -- -

If student demonstrates consistent, positive behaviors, you might disclose some of your fears

If student demonstrates repeated negative behaviors, you will either become frustrated or try to shut down the conversation.

You are the same character throughout all encounters, but you should give some different information to each student and vary your reaction slightly with each student, depending on what emotion they evoke from you. Save some of your material for subsequent students.

RELIEF THAT YOU FEEL

SUPPORTED, ONLY SLIGHTLY

VISIBLY SAD, BUT FEELING SAD,

GUILTY, TRYING TO STAY STRONG

IRRITATION / FRUSTRATION/SHUT DOWN

**REFERENCES**

Berkowitz I, Morrison W. “Do not attempt resuscitation orders in pediatrics.” Pediatr Clin N Am 54 (2007) 757–771.

Hinds P, et al. “Trying to be a good parent” as defined by interviews with parents who made phase I, terminal care, and resuscitation decisions for their children.” J Clin Oncol 27:5979-5985.

Tulsky JA. “Beyond advance directives: Importance of communication skills at the end of life.” JAMA 2005;294:359-65.

Roter DL, et al. “Experts practice what they preach.” Arch Intern Med 2000;160:3477-85.

Quill TE. “Initiating end-of-life discussions with seriously ill patients.” JAMA 2000;284: 2502-7.

Tulsky JA, et al. “Opening the black box: How do physicians communicate about advance directives?” Ann Intern Med 1998;129:441-9.

Tulsky JA, et al. “How do medical residents discuss resuscitation with patients?” J Gen Intern Med 1995;10:436-42.

JHU EOL I Adult and Peds Ward Cases SP Feedback Topics Student _________

Open feedback by stating, “This is the time I share with you what it was like to be your patient.” Then ask, “What items would you like me to address?”

| **Item** | **+ Observed Behavior/Reaction/Impact on communication –** |
| --- | --- |
| Gives you time to absorb and to speak (looking at you, not rushing to fill any silence.) |  |
| Empathetic, legitimizes your feelings and reactions |  |
| Compassionately listens, allows time to think and ask questions |  |
| Recognizes and responds to your emotional signals |  |
| Builds trust by acknowledging conversation is awkward, expresses partnership with you, makes effort to get to know you as a person |  |
| Legitimizes your feelings |  |
| Offers support |  |
| Explores statements you make or emotions you display |  |
| Expresses terms in clear simple language |  |
| Assesses your goals |  |
| Explains each, and asks if you have, a living will, advance directive, or healthcare proxy |  |
| Asks you your understanding of your condition |  |
| Provides you with the appropriate medical information, CPR, ventilator |  |
