# Supplementary material for: End-of-Life Care: A Multimodal and Comprehensive Curriculum for Graduating Medical Students Utilizing Experiential Learning Opportunities
Source: MedEdPORTAL. 2021 Apr 27;17:11149. doi: 10.15766/mep_2374-8265.11149 (PMC8076371; doi:10.15766/mep_2374-8265.11149)
Supplement: Supplementary file 1 — End-of-Life 1 Faculty Guide.docxEnd-of-Life 1 Student Handouts.docEnd-of-Life 1 Standardized Patient Materials.docxEnd-of-Life 2 PowerPoint Presentation.pptEnd-of-Life 2 Faculty Guide.docxEnd-of-Life 2 Simulation Materials.docxEnd-of-Life 2 Simulation Case Faculty Guide.docxEnd-of-Life 2 Standardized Patient Materials.docxEnd-of-Life Assessment.docx [file mep_2374-8265.11149-s001.zip › E. End-of-Life 2 Faculty Guide.docx]

**Problems in Death Certification PowerPoint Guide for Lead Facilitator**

*****A copy of the United States Standard Certificate of Death (along with link to free source) is included at the end of this faculty guide (see slide 1 note and slide 4 for our institutions state form). However, it will be beneficial to use your own local form.

Students are invited to ask questions throughout the session but there is also time at the end.

Slide 1: The lead facilitator hands out blank copies of a death certificate and explains the basic format of the Part I cause of death statement (most proximate cause to underlying cause with “due to or as a consequence of) between each line, up to four lines. Successive individuals in the class then read out each paragraph of the included patient history. Students then divide into groups of three or four and write down their formulations for a cause of death statement for the patient on the board, given approximately 10 minutes to do so. The statements are read out without comment as to accuracy and the PowerPoint presentation then begins. The activity engages learners and makes important points about the inaccuracy of death certificates which are filled out by untrained caregivers.

Slide 2: Why do we care about death certificates? They are definitely and actively used. Read out uses, short and long term.

Slide 3: Acknowledgment that some death certification is electronic, but same guidelines apply.

Slide 4: An example death certificate – exact forms will vary across states, but basic rules and formats are the same. Be sure to follow instructions!

Slide 5: Review common simple mistakes.

Slide 6: Explain that students can only certify natural deaths. Any death with another manner – homicide, suicide, accident, unknown – must be reported to the Medical Examiner, even if the underlying cause is remote in time. Medical Examiner will not necessarily do an autopsy themselves but may co-sign the certificate for a death that is not natural. More on manner of death later.

Slide 7: Was an autopsy performed? Remember that death certificate will often be filled out prior to the autopsy, but it can and should be amended if the autopsy shows something different from what was filled out.

Slide 8: Run down points one by one. Be sure to pause for effect.

Slide 9: It’s the law, and also the right thing to do for patients and families.

Slide 10: Manner and mechanism of death are not the same thing as cause of death. Categories listed are the only manners of death that there are. A cause of death is a disease condition – mechanism are physiologic processes. Do not use mechanisms on a death certificate!

Slide 11: Mechanisms do not work on a death certificate because they are non-specific. Review example.

Slide 12: Review of how Part I of the death certificate is formatted and written.

Slide 13: Review examples of acceptable causes of death. Remember that it is perfectly acceptable to put “probable” next to a cause and this is far better than putting a cause that is not useful.

Slide 14: Review unacceptable examples – there are lots of correct ways to fill out a death certificate, but what students want to do is avoid incorrect ways. Use usefulness as a gauge – what would caregivers, researchers, the state want to know? Watch out for terms that might suggest a non-natural cause -i.e. hemorrhage without an explanation.

Slide 15: This is one formulation for the cause of death statement for the history reviewed at the beginning of the session. Review, reminding students that they should maximize useful information provided.

Slide 16: Completion of cause of death statement.

Slide 17: What if underlying cause of death was different (MVA instead of MS). Ask group what manner of death is and if they could sign death certificate. They could not, because manner of death is accident (trauma) and ME would need to be called.

Slide 18: Another example – cite importance of providing underlying cause to differentiate between different possible causes of same disorder, as MI.

Slide 19: Diagnosing cause of death is the same process as diagnosing disease in a living patient – put together history, physical exam, laboratory and imaging information and arrive at best diagnostic inference. Death certification is not a separate or foreign process.

Slide 20: Example of single line cause of death, which is fine to do if there is no more information or no other underlying cause.

Slide 21: Explanation of Part II of the death certificate, which consists of contributing conditions not directly in the chain of circumstances in Part I. May be risk factors for Part I disease or entirely unrelated, not listed in any order.

Slide 22-23: Read examples of completed death certificates including Part II.

Slide 24: Facilitator reads example patient history and asks group to formulate Part I of the patient’s death certificate.

Slide 25: Read out one acceptable formation for this patient. Potential issue would be if “abdominal hemorrhage” was put down without listing underlying causes – ME might be alerted to a potential trauma, i.e. non-natural death.

Slide 26: Read out humorous incorrect examples of death certificates.

Slide 27: Final review of important points in death certification.

Slide 28: Time for final questions.

Common questions include guidelines for fetuses (in most states, a certificate issued for fetuses over 20 weeks gestational age), what to do when patient information is limited (see slide 19), reviewing examples.

**DEATH CERTIFICATE CASE**

**INTERN TRANSFER (OFF-SERVICE) NOTE**

Patient: John Andrews

DOB: 7/8/58

MRN: 123-45-678

Hospital course:

JA was a 49-year-old Caucasian man with known multiple sclerosis for 8 years complicated by paraplegia with limited use of his arms, and chronic decubitus ulcers. Five days prior to admission, he was admitted to the intensive care unit with high-grade fevers, chills, and rigors, and leukocytosis (19 × 103/μL) with bandemia of 58%.

Vital signs included the following: temperature, 102.5°F; pulse, 128 bpm; BP, 85/55 mmHg; and oxygen saturation, 96% on room air.

He also had a chronic indwelling urinary catheter, which had been recently changed. Urinalysis revealed gross pyuria and bacteriuria. Urine and blood cultures were obtained. He was started on levofloxacin (500 mg once daily intravenously) and was given 1.5 L of normal saline, after which his blood pressure improved to 115/60 mmHg. He was continued on normal saline at 100 cc/hr. He was stable for the next 12 hours when his blood pressure dropped to 60/40 mmHg. At that time, he was started on norepinephrine to titrate to systolic blood pressure >80 mmHg. During the next 36 hours, systolic blood pressure stabilized at 80 to 90 mmHg. However, the serum creatinine rose from 1.4 to 4.6 mg/dL and serum potassium levels increased from 3.6 to 7.3 mEq/L. Kayexalate per an NG tube was given to address the high serum potassium level.

Oxygen saturation dropped to 79% on room air, and he was subsequently put on

a 100% oxygen through a non-rebreather mask. Blood pressure started to decrease, and telemetry showed intermittent 20 beat runs of non-sustained monomorphic ventricular tachycardia.

Mr. Andrews had previously completed advance directives and discussed with his family that he did not desire CPR or electrical conversion of arrhythmias or mechanical ventilation. He stated that he would accept chemical intervention for hemodynamic disturbances.

On the night prior to his death he was given an amiodarone bolus and started on an amiodarone drip. He remained in normal sinus rhythm with multiple frequent premature ventricular contractions and a blood pressure of 60/30 mmHg. Norepinephrine was increased, and dopamine was started. Despite this, his systolic blood pressure did not rise above 75.

He was not responsive and eventually entered asystole and was pronounced dead.

Past Medical History

1. Multiple sclerosis (primary progressive) x 8 years with paraplegia
2. Chronic urinary retention due to MS with chronic Foley catheter
3. Stage IV sacral and bilateral greater trochanter decubitus ulcers
4. Asthma, mild persistent
5. Atopic dermatitis

Meds

Norepinephrine gtt Kayexelate

Amiodarone gtt Baclofen

Dopamine gtt

Levofloxacin

**Autopsy Basics PowerPoint Guide for Lead Facilitator**

Students are invited to ask questions throughout the session but there is also time at the end.

Slide 30: Introduction.

Slide 31: Woodcut from 1751 of a judicial autopsy. Point out judge presiding, criminal having autopsy done, intestines streaming onto floor, dog eating heart, bone boiling – trigger animation to cross out picture. Picture is included to point out that autopsy today is not this kind of grotesque scene. Autopsy is a diagnostic medical procedure done for a patient, not something we do to a body. I personally try to use the term “patient” with trainees and others and not say “body” – this attitude is important. Bereaved families are also our patients.

Slide 32: We should not do autopsies only because we always have – there are many reasons to do them and unexpected causes of death is only the tip of that iceberg. Autopsies do still discover major unexpected findings related to cause of death 5 to 8% of the time and other major and minor findings even more often. However, here are many other important reasons to perform them. Even in a patient dying of known metastatic cancer there is much to learn -what effect did treatment have, what infections did patient get? Also helps families tremendously with closure – when patients put on comfort care at the last, far from not wanting the invasive procedure, families are given comfort in their final decision by documentation of the lethality of the disease.

Slide 33: Classic 2004 study looking at autopsy opinions. Opinion of physicians/caregivers is most important independent variable to whether an autopsy was consented to.

Slide 34: As opposed to a forensic autopsy, a hospital autopsy must have a consent. Signer of the consent can decide whether to restrict procedure, but a full autopsy will not affect a viewing (no face, no limbs that will show).

Slide 35: Example of Autopsy service logistics – could be substituted for facilitator’s center.

Slide 36: Review of who can give consent for autopsy. This will vary by state, though in states where must be legal next of kin the order given usually applies.

Slide 37: Important to know that POA ends with death. Give example of a son there in the hospital making all medical decisions for father including to put on comfort care – but if there is a spouse, son may not sign autopsy consent, spouse must.

Following slides are a review of components of autopsy consenting. Though exact practices may vary by center, basic principles should apply everywhere.

Slide 38: Read through importance of patient identification on consent and on patient.

Slide 39: Autopsy consent will indicate relationship of signer to deceased patient – caution students to read this section carefully for accuracy.

Slide 40: Read through examples of figuring out who legal next-of-kin should be by state-mandated order.

Slide 41: Legal next-of-kin may restrict the autopsy procedure in any way that they wish. It will be much easier for the autopsy prosector if a body region (i.e. chest only, chest and abdomen) is specified rather than trying to list out organs (esophagus, stomach, pancreas) as listing creates dilemmas during dissection (leave aorta behind?). Can also specify areas to avoid – i.e. “no head”.

Slide 42: It is important to convey the value of a complete autopsy to families – remember that limited autopsy may mean questions are not addressed. Caution students not to assume they know what happened, as “patient died of a heart attack so heart only will address questions.” Literature shows that unexpected causes happen, also that doctors are not able to effectively select which cases these will be. We do not know what we do not know. That said, wishes of family will always be respected.

Slide 43: Review other areas that autopsy consent may cover – retention of tissues and their use.

Slide 44: Review details about signatures. Bear in mind that in many states consent must be signed after the patient’s death.

Slide 45: Review other details and indications that autopsy consent may have.

Slide 46: Study about stakeholder perceptions of autopsies performed for research – note disconnect between physician thoughts about patient discomfort being the greatest difficulty while families think physicians are worried about logistics. We need to serve the deceased patients and families and not assume that we know what they think about autopsy. Everyone should be asked.

Slide 47: The Final Pearl. Remind learners that autopsies are important and valuable for many reasons. They can provide unique opportunities in clinical care, education, and research. It is up to healthcare providers to make this happen for patients, families, and physicians.

Time for questions.

<https://www.cdc.gov/nchs/data/dvs/death11-03final-acc.pdf>
