# Supplementary material for: End-of-Life Care: A Multimodal and Comprehensive Curriculum for Graduating Medical Students Utilizing Experiential Learning Opportunities
Source: MedEdPORTAL. 2021 Apr 27;17:11149. doi: 10.15766/mep_2374-8265.11149 (PMC8076371; doi:10.15766/mep_2374-8265.11149)
Supplement: Supplementary file 1 — End-of-Life 1 Faculty Guide.docxEnd-of-Life 1 Student Handouts.docEnd-of-Life 1 Standardized Patient Materials.docxEnd-of-Life 2 PowerPoint Presentation.pptEnd-of-Life 2 Faculty Guide.docxEnd-of-Life 2 Simulation Materials.docxEnd-of-Life 2 Simulation Case Faculty Guide.docxEnd-of-Life 2 Standardized Patient Materials.docxEnd-of-Life Assessment.docx [file mep_2374-8265.11149-s001.zip › F. End-of-Life 2 Simulation Materials.docx]

| **Appendix F: MedEdPORTAL Simulation Case Template**  **SIMULATION CASE TITLE: Adult Cardiac Arrest and Death Exam**  **AUTHORS: TRIPLE Faculty**  **LEARNER AUDIENCE: Graduating Medical Students** | |
| --- | --- |
| **PATIENT NAME: Mr. Singler**  **PATIENT AGE: 73**  **CHIEF COMPLAINT: Unresponsive**  **PHYSICAL SETTING: Inpatient med/surg floor** | |
|  | |
| **Brief narrative description of case** | 73-year old male admitted for endocarditis (non-surgical). Learners will find patient in asystolic cardiac arrest. Patient will remain in cardiac arrest for the duration of the case.  Learner Goals:   1. Manage asystolic cardiac arrest using Advanced Cardiac Life Support (ACLS) guidelines 2. Perform an appropriate and complete death exam |
| **Primary Learning Objectives** | 1. Appropriately manage asystolic cardiac arrest per ACLS guidelines 2. Appropriately end a resuscitation 3. Adequately perform a death exam per faculty guide |
| **Critical Actions** | 1. Assess patient and recognize pulseless state 2. Immediately begin CPR and begin to organize the team 3. Implement appropriate crisis resource management skills such as closed loop communication, situational awareness, shared mental model, etc. 4. Perform excellent cardiopulmonary resuscitation based on ACLS guidelines 5. Recognize futility of continued efforts and end the resuscitation 6. Perform a death exam |
| **Learner Preparation or Prework** | It is important to recognize this case was developed for learners who have advanced life support training. If learners do not have advanced life support training, the case may be altered to accommodate. One way to do so would have learners enter the room as the faculty leader is ending the resuscitation. Or have the faculty lead function as the team leader directing the learners. Both options may shorten the duration of the case.  Learners will be given a progress note for the patient just prior to entering the room. This progress note is included in this appendix |

| Initial Presentation | | | |
| --- | --- | --- | --- |
| **Initial vital signs** | HR 0, asystole, pulseless, RR 0, Pulse oximetry 0%, Blood Pressure unable to obtain. Temperature 37.2 C | | |
| **Overall Setting and Appearance** | Learners enter a standard inpatient room with cardiac monitoring. They should see the patient laying in a hospital bed in a gown covered with a blanket. They should also see the patient cardiac monitor with the above vital signs and heart rhythm. Mannequin should be laying with eyes closed, not breathing. | | |
| **Confederates (e.g., standardized participants) and their roles in the room at case start** | The confederate will be playing the role of nurse and should have the skills to function at a very basic level. For example, place peripheral IV catheter, draw blood, give fluids and medications. This role can be played by the faculty member who will discuss the death exam with the students and debrief the case but may also be played by anyone who fits the description.  Initial script is: Call students into room and introduce themselves. State “Mr. Singler doesn’t seem to be waking up. Can you assess him?”  Additional scripts are undefined but include answering patient history questions when asked and agree to perform asked tasks.  If learners need prompting to end the resuscitation, confederate may say things like, “I think we’ve done all we can” or “How much longer would you like to resuscitate?” | | |
| **HPI** | Please see student handout included in this appendix | | |
| **Past Medical/Surgical History**  ASKED: Past history: coronary artery disease, myocardial infarction (15% ejection fraction),insulin dependent diabetes mellitus, chronic renal insufficiency, atrial fibrillation, dementia.  **Problem List:** 1. Endocarditis with new congestive heart failure. Patient is not a surgical candidate.  2. Insulin dependent diabetes mellitus  3. Delirium/dementia – confused, disoriented  4. Chronic kidney disease. Recent increase in creatinine, likely from new heart failure | **Medications**  Vancomycin, rifampin, lisinopril, metoprolol, aspirin, furosemide, simvastatin, insulin, glipizide | **Allergies**  **NKDA** | **Family History**  Cardiac disease, hypertension, diabetes, numerous forms of cancer |
|  |  |  |  |
| **Physical Examination** | | | |
| **General** | Not moving and unresponsive | | |
| **HEENT** | Pupils not reactive, otherwise normal | | |
| **Neck** | Supple, no obvious abnormalities | | |
| **Lungs** | Not breathing. Lungs clear with bag-mask ventilation | | |
| **Cardiovascular** | Pulseless | | |
| **Abdomen** | Soft, non-tender, non-distended | | |
| **Neurological** | Unresponsive | | |
| **Skin** | Pale, very delayed capillary refill time | | |
| **GU** | Normal gender appropriate genitalia | | |
| **Psychiatric** | History of delirium. Currently unresponsive | | |

| Instructor Notes - Changes and CASE Branch Points | | |
| --- | --- | --- |
| **Intervention / Time point** | **Change in Case** | **Additional Information** |
| Time(T)=0 minutes | *Patient unresponsive in cardiac arrest* | *RN alerts the provider: Patient won’t wake up. Can you assess him?* |
| *T=1 minute* |  | *Learners begin cardiac arrest management, start chest compressions, organize the team, begin advanced life support (ALS)* |
| *T=5 minutes* | *Patient remains in asystole* | Learners continue ALS with high quality cardiopulmonary resuscitation, IV/IO access, administer epinephrine every 3-5 minutes. |
| *T=15 minutes* | *Patient remains in asystole* | *Begin to wrap up resuscitation. Leader gives summary of events, asks for any other additional information, ideas, or suggestions. End resuscitation.* |
| *T=20 minutes* | *Debriefing and education* | *Faculty lead a brief debriefing of the medical events followed by a longer discussion and practice opportunity for performing a death exam* |

**Ideal Scenario Flow**

The learners enter the room to find the patient unresponsive and in asystole. They immediately begin CPR, ask for defibrillator, backboard, stool, code cart, and for airway equipment to be set up. They then organize the team into roles including team leader, airway, compressor (x 2 if possible), medication nurse, and other roles depending on available numbers. Once team roles are established, learners inquire about relevant past and current history that may have led to cardiac arrest. High quality CPR continues throughout based on ACLS guidelines including appropriate rate and depth, ratio of compressions to breaths, utilizing end tidal Co2 and a feedback device (most defibrillators) to monitor CPR quality, epinephrine every 3-5 minutes, and appropriate team dynamics. Labs may be sent but will not return during the resuscitation except for a bedside glucose, which will be normal. Advanced response teams may be asked for but will not arrive during the resuscitation. Imaging may be asked for but will not be completed during the resuscitation. After 15-20 minutes, learners will end the resuscitation. If needed, the confederate will prompt them to do so.

**Anticipated Management Mistakes**

1. *Delay in recognizing cardiac arrest and initiating CPR: We found learners may get distracted by non-relevant initial details such as patient history or medications instead of immediately initiating CPR.*
2. *Difficulty ending the resuscitation: Many learners are uncomfortable stopping a resuscitation and/or don’t know how to end it. We found having the confederate give a kind, but purposeful prompt helped.*

**GIVE TO STUDENTS PRIOR TO SIMULATION CASE**

**Brief Patient Summary**

| Name/MRN: Bob Singler 1234567, age 73 |
| --- |
| Attending: Garibaldi |
| Code status: FULL |
| All: Sulfa |
| Meds: vancomycin; rifampin; lisinopril; metoprolol; aspirin; furosemide; simvastatin; Lantus, sliding scale; glipizide; |
| PMH: Coronary artery disease status post myocardial infarction; ischemic cardiomyopathy, ejection fraction 15%; type 2 diabetes mellitus; chronic renal insufficiency; atrial fibrillation; dementia |
| Problems:   1. MRSA endocarditis - Has new onset severe aortic regurgitation from valvular damage with CHF in setting of endocarditis. Being treated with vancomycin, but only curative treatment is valve replacement. Surgery consulted but thinks pt too chronically ill to have surgery. Family meeting yesterday, offered hospice, but family unable to make a decision yet. 2. Type 2 diabetes mellitus-on Lantus/sliding scale, oral intake poor 3. Delirium/dementia-confused, oriented to person only; sleeping a lot 4. Chronic kidney disease - creatinine worse than baseline, likely from CHF |

**Intensive Care Progress Note**

Bob Singler #1234567

S: No overnight events. Poor oral intake. Patient denied complaints, still confused. Family meeting yesterday, no decision made about hospice.

O: Gen: drowsy, arousable

VS: BP 85-100/40-50 HR 80-90 RR 20 O2 Sat 92% on 2Liters nasal canula T 37

I/O: 1200ml/2000ml

CV: irregular rate and rhythm; s2; II/VI diastolic murmur; +jugular venous distention

Lungs: bilateral crackles

Abd: soft, non-tender, non-distended

Ext: 2+ edema to mid shin

Neuro: inattentive, disoriented

AM Labs:

133 100 50 100 17 8.4 380

4.2 22 2.2 24.8

Chest X-Ray: pulmonary edema

Blood culture 4/6: pending

Blood culture 4/5: methicillin resistant Staphylococcus Aureus

Blood culture: 4/4: methicillin resistant Staphylococcus Aureus

EKG: atrial fibrillation, left bundle branch block, Qs in II, III, aVf

Meds

vancomycin; lisinopril; metoprolol; ASA; furosemide; simvastatin; Lantus, sliding scale; glipizide

A/P:

72 yo man with MRSA endocarditis, severe AI, CHF

1. Cardiovascular: Infective endocarditis (MRSA), with severe AI. Remains on vancomycin day#6 with persistently positive blood cultures. Clinically no improvement. Still volume overloaded with no improvement on diuretics. BP did not tolerate higher dose furosemide. On very low dose angiotensin-converting-enzyme inhibitor and beta blocker. Transthoracic echocardiogram done 4/4 showed vegetation on aortic valve and severe aortic insufficiency. Question of a perivalvular abscess. Only curative treatment is valve replacement. Surgery consulted but thinks patient too ill to have surgery.
2. Renal: Baseline creatinine 1.5, currently 2.3. May be from heart failure or possibly septic emboli
3. Endo: DM 2, sugars controlled on Lantus, SSI
4. Neuro: has baseline dementia, also delirious
5. Pulm: oxygen saturation acceptable on 2L NC

Dispo: Family meeting yesterday to discuss poor prognosis, surgery not an option. Hospice offered. Family not in agreement about the plan. For now, remains full code and in ICU.
