# Supplementary material for: End-of-Life Care: A Multimodal and Comprehensive Curriculum for Graduating Medical Students Utilizing Experiential Learning Opportunities
Source: MedEdPORTAL. 2021 Apr 27;17:11149. doi: 10.15766/mep_2374-8265.11149 (PMC8076371; doi:10.15766/mep_2374-8265.11149)
Supplement: Supplementary file 1 — End-of-Life 1 Faculty Guide.docxEnd-of-Life 1 Student Handouts.docEnd-of-Life 1 Standardized Patient Materials.docxEnd-of-Life 2 PowerPoint Presentation.pptEnd-of-Life 2 Faculty Guide.docxEnd-of-Life 2 Simulation Materials.docxEnd-of-Life 2 Simulation Case Faculty Guide.docxEnd-of-Life 2 Standardized Patient Materials.docxEnd-of-Life Assessment.docx [file mep_2374-8265.11149-s001.zip › G. End-of-Life 2 Simulation Case Faculty Guide.docx]

**TRIPLE Death Pronouncement/Death Notification Faculty Guide**

This session is one of three stations during a half-day session designed to train students in skills necessary at the time of a patient death. These skills include death pronouncement, notification of family of a death via standardized patient (SP) encounter, completing death certificates, discussing autopsies and organ donation.

Overall objectives for the session include (relevant objectives for this station in bold):

By the end of the session, the student will be able to (bold indicates objectives specific to this station):

1. **Demonstrate proper method of pronouncing a person to be dead**
2. **Inform a family member of a death in a clear and compassionate manner**
3. Correctly complete a death certificate
4. Answer frequently asked questions about autopsies
5. Describe how to approach a family member about consent for an autopsy of their deceased family member
6. Describe appropriate approaches to discussing organ donation with families

There will be seven-eight students in your group.

**General break down of timing:**

| 0-20 mins | Code |
| --- | --- |
| 20-35 mins | Debriefing and Death Pronouncement (DP) |
| 35-45 mins | SP Prep |
| 45-50 mins | Transition to SP room |
| 50-60 mins | SP Encounter *(Faculty break)* |
| 60-75 mins | SP Feedback *(Faculty break)* |

**Overall Group Assignments:**

8:00-8:15 AM ALL GROUPS – Orientation in Sim Center Classroom

| **TIME** | **STATION I**  Death Certificate | **STATION II**  Autopsy/Organ Donation | **STATION III**  Code, DP, SP Case | **BREAK** |
| --- | --- | --- | --- | --- |
| 8:15-9:15 AM | Group 1 | Group 2 | Group 3 A, B, C | --- |
| 9:15-9:30 AM |  | --- |  | Group 2 |
| 9:30-10:30 AM | Group 3 | Group 1 | Group 2 A, B, C | --- |
| 10:30-10:45 AM |  | --- |  | Group 1 |
| 10:45-11:45 AM | Group 2 | Group 3 | Group 1 A, B, C | --- |
| 11:45-12:00 AM |  | --- |  | Group 3 |

**Station III A, B, C - Death Pronouncement and Notification:**

Specific times of segments

| ALL GROUPS | Orientation | 15 mins | 8:00 AM | 8:15 AM |
| --- | --- | --- | --- | --- |
| GROUP 3  A, B, C | Code, DP, SP Prep, Transition | 50 mins | 8:15 AM | 9:05 AM |
|  | SP Encounter | 10 mins | 9:05 AM | 9:15 AM |
|  | SP Feedback prep and delivery | 15 mins | 9:15 AM | 9:30 AM |
| GROUP 2  A, B, C | Code, DP, SP Prep, Transition | 50 mins | 9:30 AM | 10:20 AM |
|  | SP Encounter | 10 mins | 10:20 AM | 10:30 AM |
|  | SP Feedback prep and delivery | 15 mins | 10:30 AM | 10:45 AM |
| GROUP 1  A, B, C | Code, DP, SP Prep, Transition | 50 mins | 10:45 AM | 11:35 AM |
|  | SP Encounter | 10 mins | 11:35 AM | 11:45 AM |
|  | SP Feedback prep and delivery | 13 mins | 11:45 AM | 12:00 PM |

**Pre-Brief (A conversation to be had with learners prior to starting the case)**

The case you are about to participate in involves a patient in cardiac arrest and who may not survive. I understand some of you may have experience with death both personally and professionally. Those past experience may impact your experience today. Even though this is a simulated environment, it is natural to feel stress, anxiety, and a variety of other feelings during the simulation. If at any time you feel you cannot continue, that is your decision, and it will be fully supported. If at any time (during the case or after), you would like resources, they will be provided to you.

(Institution specific resources should be available)

**Code/resuscitation**

The setting: Medicine floor

Students are the intensive care unit team of the day, responsible for responding to codes. Faculty should tell/remind the learners of this during the pre-brief. They will be told by the “nurse” that a patient just went into asystole. Faculty should give the learners three types of information about the patient prior to entering the simulation room: a sign-out sheet, the morning labs, and the last progress note written about the patient (Appendix F). The patient is a “full” code and they will be expected to work as a team to run through the asystole algorithm.

Your role: Facilitator one: You serve as both the “nurse” and the supervising fellow.

Facilitator two: Operates the manikin.

The following sequence describes the case in the setting of only having one facilitator. If two facilitators, one operates the manikin and the other remains in the simulation room and serve as nurse confederate. Then at the end of the case (as described below), the control room facilitator may enter the room as the senior provider.

**Asystole Resuscitation Sequence:**

Students will enter the room and you will announce over the intercom to the group, “Thank goodness you’re here doctors, this patient’s heart just stopped.” Specific medical information about “the patient” is on the next two pages.

You will announce through the microphone in the control room that looks into the simulation room as the students enter.

Students will be expected to assess the patient (a high-fidelity sim-man, but any high-fidelity manikin will suffice) and begin the advanced cardiac life support (ACLS) algorithm. There will be a crash cart with “drugs” available as needed. Regardless of the intervention, the patient will have no response in electrical activity or vital signs. You will not have to worry about changing manikin settings for that reason.

Students may ask questions that you will be able to hear through the headphones in the control room, asking for info from the “nurse” or asking for a consult. You can respond as you see fit, if you need to make up info that is okay too! Just be sure it doesn’t lead to the patient living through the scenario. The most important thing is that they just follow the basic algorithm.

Students may ask if the “nurse” can call a fellow or attending. Say “okay, they are on their way”. Whether the students ask for a fellow or not, after nine-ten minutes, you should leave the control room and walk into the simulation room as “the fellow”.

You will ask the students to briefly update you on what is going and then you will instruct the students to stop resuscitation efforts as they are almost certainly futile for this patient.

You will then transition on to the teaching points. See below.

**Teaching Points:**

1. **Debrief resuscitation**

Please only spend a couple of minutes on this. If you saw any glaring deviation from the Advanced Cardiac Life Support algorithm, discuss that with them. They will have had plenty of chance to practice resuscitation before today and I want to be sure you have time to cover the death pronouncement aspects. **However, an important teaching point may be how a team leader decides when to stop resuscitation efforts.**  In previous practice, the students get to keep trying until the patient lives. They have not practiced any resuscitation this week in which the patient actually dies.

1. **Discuss death pronouncement**
   1. Death pronouncement after a code

What is required for a death exam?

Must determine that there is absence of cardiopulmonary and neurologic activity.

That is done by:

- Identify the patient by the hospital ID tag. Note the general appearance of the body.
- Ascertain that the patient does not rouse to verbal or tactile stimuli. Listen for the absence of heart sounds at least 30 seconds; feel for the absence of carotid pulse at least 30 seconds
- Look and listen for the absence of spontaneous respirations, at least 30-60 seconds.
- Record the position of the pupils and the absence of pupillary light reflex.
- Record the time at which your assessment was completed.
- DO NOT: Use overtly painful stimuli, especially if family members are present. Nipple or testicle twisting, or deep sternal pressure are inappropriate and unnecessary.
  1. Have the appropriate people been called? Was the attending notified? The primary care physician?
     - A frequently asked question (FAQ) is whether you should call attending in the middle of the night when a patient dies. You may want to address that (the answer for most attendings is yes, unless it is a very expected death)
  2. How will the family be notified?
     - An FAQ is whether families should be informed over the phone (see number 3 below).
     - An FAQ is what happens if the body twitches or emits a noise? This can be normal, and short lived.
     - An FAQ is what to do with pacers (especially if still spikes on telemetry despite no pulse and failed resuscitation) and defibrillators. They can be turned off with a magnet, but they don’t have to be.

1. **Delay vs. Disclose Immediately**Few would disagree that it is always preferable to present death notification in person. However, when families live at a great distance, or are physically unable to travel, telephone discussion will be necessary. In other situations, clinicians must weigh the benefits of truthfulness against the risk of potential harm resulting from abrupt disclosure of the bad news. Factors to consider in making this decision include:

- Whether death was expected or not, including the nature and chronicity of the illness.
- How well the death notifier knows the patient and the patient’s family.
- The relationship of the contact person to the patient.
- The anticipated emotional reaction of the contact person based on prior information.
- Whether the contact person will be alone when receiving the information
- The contact person's level of understanding.
- Distance, availability of transport, and time of day.
- If you decide to delay disclosure of the death, be prepared to make immediate admission of such nondisclosure as soon as you meet the family (e.g. I'm sorry for not telling you the whole thing right away over the phone; then give your reason for doing so).

1. **Documentation in the Medical Record**

- Called to pronounce (name); Chart the findings of physical examination.
- Note date and time of death; Note if family and attending physician were notified.
- Document if family declines or accepts autopsy; document if the coroner was notified.

1. **Death pronouncement of an unwitnessed inpatient death**

Some additional steps necessary:

**The Phone Call: “Please come and pronounce this patient”**

- Find out the circumstances of the death – expected or sudden? Is the family present?

**Preparation Before You Enter the Room**

- Get the details on the circumstances of death from the nurse.
- For residents, find out if the attending physician has been called. In general, see the patient before calling the attending, unless there are unusual family dynamics or details surrounding the death that you should discuss with the attending.
- Determine if the family has requested or if you believe there is value in requesting an autopsy. Some institutions have specific policies about autopsy requests.
- Determine if the patient/family has already been contacted by the Organ Donor Network
- Review the chart for important medical (length of admission, cause of death) and family issues (Who is family? Faith? Is there a clergy contact?).

**In the Room**

- You may want to ask the nurse or chaplain to accompany you; he/she can give you support and introduce you to the family.
- Introduce yourself (including your relationship to the patient) to the family. Ask each person their name and relationship to the patient.
- Empathetic statements are appropriate: “I’m sorry for your loss...” Or – “This must be very difficult for you....”
- Explain what you are there to do. Tell the family they are welcome to stay while you examine their loved one.
- Ask if family members have any questions or if they wish to speak with a chaplain.
- Perform the death exam
  - A FAQ is whether families should stay in the room for the death exam. Typically, a provider will explain the process and give the family the option to leave. You can share whatever your practice is.

1. **Ask a few students to demonstrate the death exam on sim-man**
2. **Set up death notification scenario**

- Tell students they should break up into pairs. If you have an odd number, one student will be solo.
- Tell students that the nurse told you that a family member of Mr. Singler’s has just arrived and wants to know what’s going on.
- Acknowledge that residents are sometimes put into uncomfortable situations of notifying a family member of a death when they have never met them.
- Tell students they will go in pairs to discuss this. It is also not uncommon to have another team member present for these discussions.
- You may share some of your own wisdom in these scenarios. ***You may also discuss when to disclose over the phone vs. when to ask family to come in and notify in person.***
- Pass out the handout on death notification/breaking bad news.
- Tell students they have five minutes to strategize between them how they will handle the conversation and think about what they will say.
- Send students out to the area just outside the sim room.
