# Supplementary material for: End-of-Life Care: A Multimodal and Comprehensive Curriculum for Graduating Medical Students Utilizing Experiential Learning Opportunities
Source: MedEdPORTAL. 2021 Apr 27;17:11149. doi: 10.15766/mep_2374-8265.11149 (PMC8076371; doi:10.15766/mep_2374-8265.11149)
Supplement: Supplementary file 1 — End-of-Life 1 Faculty Guide.docxEnd-of-Life 1 Student Handouts.docEnd-of-Life 1 Standardized Patient Materials.docxEnd-of-Life 2 PowerPoint Presentation.pptEnd-of-Life 2 Faculty Guide.docxEnd-of-Life 2 Simulation Materials.docxEnd-of-Life 2 Simulation Case Faculty Guide.docxEnd-of-Life 2 Standardized Patient Materials.docxEnd-of-Life Assessment.docx [file mep_2374-8265.11149-s001.zip › H. End-of-Life 2 Standardized Patient Materials.docx]

**EOL II Bob Singler (with simulated survivor Chris)**

# CASE SUMMARY

**OVERVIEW**

**Objective:**

To assess fourth year medical students’ skills in delivering news of the sudden death of the father (brother) or brother of an adult via a standardized patient (SP) encounter.

**Competencies Assessed:**

1. Patient education and counseling skills

2. Negotiation and shared decision-making skills

3. Relationship development skills

4. Global satisfaction

**Logistics**:

**Personnel**- SP, can be adapted for any age approximately 30-50, sitting in chair

Small group exercises, one or two learners

**Station Materials:**

1. Instructions for the examinee

2. SP instructions

4. Evaluation forms

**Room arrangement:**

1. Door sign (or some other way to direct students to correct room)

2. Outpatient exam table pushed against wall

3. Chair for SP

4. Chair/stool for doctor(s)

**STANDARDIZED PATIENT INSTRUCTIONS**

**Chris Singler** (child of patient if approximately 35-55 years old, sibling if approximately 55-75 years old and need to adjust scenario)

**The Scenario**

Time: morning

Your 72-year-old father (brother) has been in the hospital for over a week. His health has not been good for several years. He had a heart attack at the age of 60 and has really never been the same since. He frequently has a buildup of fluid in his lungs and legs (aka heart failure), making it difficult for him to breathe and walk around. He also has diabetes which he didn’t take very good care of when he was younger and now has kidney problems and eye problems from it. Despite all of this, your father (brother) still was able to do some things around the house (like wood carving and washing dishes) and get out for family functions and to see his friends at the diner. He also has started to develop some memory problems in the past couple years and needs to be reminded to pay bills and take his medicines.

Your mother (sister-in-law) is 75 and in better health but does have some of her own health issues. She has taken on much of the burden of the household chores but doesn’t seem to mind.

Since age 60, he would have frequent doctors’ visits and would usually be hospitalized yearly for the fluid buildup. However, in the past 6 months he has been much sicker. He has been hospitalized 3 times and had to go to a rehab facility to regain his strength each time, although he has never really gotten back to himself. On the last admission, he was really sick and in the ICU with fluid buildup. His heart was beating fast and irregularly (atrial fibrillation). He managed to recover from that and was transferred to a rehab facility again. He initially did okay, but then a few days later he had fevers, was really confused, and looked pale. His blood pressure was low, so he was transferred back to the hospital. When he got to the hospital this most recent time, they found he had an infection on his heart valve and in his bloodstream. The doctors think a special IV he had in his neck last admission (aka a central line) got infected which led to bacteria in his blood that then stuck to his heart valve.

The bacteria have really damaged the heart valve and normally a person with this problem would have to have a major open-heart surgery to replace the valve, but the doctors have said your father (brother) is too sick for this. His heart is weak to begin with (that’s why he has heart failure so often) and his kidneys are even worse than before. He is really confused and too weak even to sit up on his own. They say he probably would not survive the operation.

This news has been upsetting to you since the doctors told this to you, your mother (sister-in-law), and sister (niece) a few days ago, but you are starting to accept it. You have been worried that your father (brother) has been suffering in recent months. He seems so weak and has repeatedly said how much he hates the hospital and rehab. He misses his home and food. He says if he has to be in these places all the time, life isn’t worth living.

The doctors recommended that you place your father (brother) in hospice. You and your sister (niece) think this is probably the best thing. Obviously you do not want your father (brother) to die, but if he is dying, you want him to be comfortable and have some dignity. You were hoping to take him home with hospice. However, your mother (sister-in-law) is reluctant to do this. She is not coping as well with the news and has not been able to accept it. She is not ready to “give up” on your father (brother) and has not accepted hospice. She believes he is a “fighter” and will pull through. You and your sister (niece) wanted to tell the doctors that your father (brother) should not have any aggressive measures if he deteriorates further (i.e. “do not resuscitate”, no CPR, no breathing tube (ventilator), no dialysis) and look into hospice services. However, your mother (sister-in-law), who is his health care power of attorney, has not agreed to this. After several conversations, you, your mom, and your sister (niece), the doctors agreed that they would give him a couple more days to improve, and if he didn’t, then you would proceed with hospice and a “do not resuscitate” order.

You saw your father (brother) last night. He was still getting the antibiotics through the IV (for the valve infection). He was still pretty confused but recognized you. He barely ate, even with coaching. The doctors said he was “stable” meaning, he was still very sick without improvement but without deterioration that day.

However, this morning, the nurse called your mother (sister-in-law) and told her he had taken a turn for the worse. You live only 5 minutes from the hospital, so you got here first. The nurse asked you to wait in the waiting room. You have been in there about 10 minutes, waiting for news. Your sister (niece) is picking up your mother (sister-in-law) and they are on their way. One or two resident doctors will come and speak with you. You have never met either of them before; they are the on-call/covering doctors. [If taking place in the afternoon, your mother (sister-in-law) had been at the hospital and went home to take a nap and change her clothes.]

You are sad but this news is not unexpected. You cognitively understand that your father (brother) has been seriously ill but were still holding out hope. You are not overwhelmed with grief in this moment, the news has not sunk in. You feel a little guilty because you didn’t want your father (brother) to suffer through CPR (which did occur), but also did not want to disrespect your mother (sister-in-law)’s wishes in this regard. It is important to you to know if he suffered or not.

**Your background:** You are 35-50 years old or 55-75 years old, the oldest of 2 children and often are the family organizer. Your sister (niece) definitely helps out, but more often than not, you are the one who goes to doctors’ visits with your parents. As your dad’s memory has been failing you have taken over the finances. You completed high school, work as a bank teller, are married and have 2 children (teenage to 20’s). You have little personal experience with death since your parents and in-laws are living.

***Father (brother)’s Past History***: High blood pressure, heart attack at age 60, recurrent heart failure, diabetes, kidney problems, high cholesterol, memory problems.

It is not necessary to know anything about his medications, other than that he took a lot.

***Father (brother)’s Social History:*** Married for 50 years to your mother (sister-in-law). They have a very loving marriage and describe each other as best friends. Retired Bethlehem steel worker. Has 2 children (you and your sister (niece)), who both live in Baltimore. Your family is pretty close, and you see your parents usually twice a week. Since your dad has been so sick, you’ve been seeing your mother (sister-in-law) more. He has 4 grandchildren who are teens-20s (you and your sister (niece) have 2 kids each). He is a pretty devout Catholic and went to church every week and was active in his church until 6 months ago. Whenever he’s in the hospital, the hospital chaplain visits him, and sometimes the priest from his parish, St. Luke’s, comes to visit.

He does not smoke now but smoked pretty heavily when you were growing up. He drinks an occasional beer. He likes wood carving and stays involved in family functions.

**The Medical Encounter**

Chris Singler:

Opening statement: “***How is he?”*** *Worried, tired, nervous. You will stand up when the doctors enter, but do not need to pace.*

You are feeling really worried and nervous. You have a sick feeling in the pit of your stomach. You are really hoping the doctors will tell you that your father (brother) is stable and you can see him. But you have a feeling they are going to give you bad news.

Maintain good eye contact. Listen carefully to what you are being told. *Be careful not to anticipate what you are going to hear.*

When you are told that your father (brother) has died, you are initially in disbelief, but then quickly become sad. You are not overtly overwhelmed with grief. You may have a few tears or need a minute to take some deep breaths but then you move into being concerned about your mother (sister-in-law) and how she will take this news.

Possible scenarios:

1. The students will tell you right away that your father (brother) has died. If this happens, after your emotional reaction, you will have a lot of questions and need to know what happened, because he seemed stable when you left the hospital last night.
2. The students will start by asking you what you know about the situation this morning. You will say that the nurse called your mother (sister-in-law) and told her he had taken a turn for the worse but didn’t give any specifics on the phone. When you last saw him, he seemed weak and confused but you were told he was “no better, no worse”.

| ***Possible student behaviors*** | ***Your response*** |
| --- | --- |
| Introduces self and role in care of your father (brother) | If introduction and role are clear, you will be able to move forward with the conversation.  If role of student in care of your father (brother) is unclear, you will interrupt student to determine this. “*I’m sorry, can you tell me who you are again?”* |
| States your father (brother) died without a warning shot or ascertaining what you knew about the situation first. | You will become more upset than you would have otherwise. You will be shocked, then sad, and maybe mildly angry because you are confused and overwhelmed. |
| Fires a warning shot and then tells you your father (brother) died (*e.g. I’m afraid I have some bad news.”).* | You will be more receptive to the information, because you will be able to brace yourself for what is coming. You will still be mildly shocked and sad. |
| Asks you what you know about your father (brother)’s condition before notifying you of his death. | This will be the most reassuring sequence as it allows you to ease into the conversation and understand the sequence of events.  *“The nurse called my mother (sister-in-law) and told her he had taken a turn for the worse but didn’t give any specifics on the phone. When I last saw him, he seemed pretty weak and confused but the doctor said he was ‘no better, no worse’.”* |
| Reviews chronology leading up to death briefly and clearly. | You will express understanding of the situation. |
| Reviews chronology leading up to death in a rambling or unclear way. | You will be confused about the situation. |
| Uses medical jargon. | You will be confused about the situation. |
| Pays attention to the words you use and mirrors them. | You will think the student is really listening to you and feel comforted. |
| Uses euphemisms when they inform you of your father (brother)’s death (e.g. *“passed on, passed away”, etc.).* | You will be briefly confused and not sure if he is actually dead. |
| States clearly that your father (brother) is dead or has died. | You will express understanding of the situation. |
| Assesses how much information you want to be given.  “Some people really do not want to be told much at all about the medical details and would rather have other family members or a friend handle the information. Other people want every detail. What is your preference?” | You want to know the basic sequence of events, but not a lot of details. You want to know if he suffered. |
| **Respond to your emotion. Examples of positive behaviors include:**   - **Acknowledge** the emotion. I can see that this is very upsetting. You seem overwhelmed by this news. - **Legitimize** (validate) the emotion. Anyone in your shoes would be upset. A lot of people would feel angry right now. - **Explore** the emotions and be sure you understand them before providing reassurance  Tell me what is most upsetting to you….   Tell me what worries you the most…   - **Empathize** I imagine it feels overwhelming. I would probably feel the same way. - **Provide support,** and **partnership**  We will work through this together. Is there anyone you would like me to call? | You feel comforted by any of these actions. Surface empathy alone will not really reassure you much (*“I’m sorry” or “This must be hard”*) If the students displays at least one other response to emotion, you will be reassured.  The most upsetting thing to you is that your father (brother) was alone when he died. You didn’t want this for him. You wanted him to die peacefully and you feel a little guilty that did not happen. Your biggest worry is that your mother (sister-in-law) will not be able to handle the news of your father (brother)’s death.  You don’t need the student to call anyone for you, but you would like them to be available to talk with your mother (sister-in-law) and sister (niece) when they arrive. |
| **Repeatedly only responds to your emotion with *“I’m sorry.”*** | This will not reassure you much. You might say, “*It wasn’t your fault.”* |
| **Allows for silence when you are emotional.** | This will calm you down as you think you have some control over the pace and that the student cares about your feelings. |
| States, “I understand what you are going through.” | This will seem insincere as you wonder how this student could understand this situation exactly. Your response is, “Did your father (brother) die like this?” |
| Touches you | Depends on how it was handled. If the student puts a hand on your hand or shoulder, and you are feeling trust, you will be comforted by this.  If the student touches your leg, it will feel uncomfortable. |
| Asks what questions you have | If student does not ask, you will be frustrated, and you will end the encounter by asking to speak to your father (brother)’s regular doctor.  If asked, your questions should not be offered up all at once, but in response to being asked repeatedly.  *“Did he suffer?”*  *“Can you speak to my mother (sister-in-law)?”*  *“Can the chaplain provide a blessing?”*  [Last rites were done a few days ago]  *“How do we handle the funeral arrangements?”*  *“Can I see him?”* ***This should be used as the close of the encounter. |
| Asked if there was anyone they could call for you | *“No, thank you, my mother (sister-in-law) and sister (niece) will be here soon.”* |
| Tells you how they can be reached when your family arrives | Express gratitude |

Some **positive behaviors** on the student’s part might include:

- Fires a warning shot before the actual death notification.
- Giving you time to absorb and to speak (looking at you, not rushing to fill any silence.)
- Showing empathy (e.g., “I am so sorry that this happened to your wife/husband; I can only imagine how you must be feeling right now.”)
- Compassionate listening (nodding, allowing you to speak, maintaining warm eye contact)
- Responds to your emotion beyond a surface gesture.
- Remaining patient, allowing you to interrupt; answering all questions that you have.
- Offering some kind of help-- to get you more information, make calls for you, set up a time for the two of you to meet with the resident (if you have made that request).

If a student demonstrates consistent **negative behaviors**, your anxiety and sadness will increase and may lead to you ending the scenario early.

Some **negative behaviors** that would lead to this:

- Rushing through the conversation
- Failing to acknowledge your emotions at all
- Failing to express empathy
- Not allowing you to ask questions or not answering your questions
- Using complex language

***To end the scenario, you can 1. Say you want some time to collect yourself before your mother (sister-in-law) arrives or 2. Ask if you can see your father (brother)’s body. You will not actually leave the room. You may ask for a few minutes to collect yourself which will allow the student(s) to exit.

**Emotional modulation:**

1. Start out—*worried, tired*

2. After hearing about your father (brother)’s death—*sadness, guilt*

3. If the doctor comforts you or responds to emotion, builds trust—*become calm and sad*

4. If the doctor fails to respond to emotion, pause, or allow for your reaction or questions—*grow frustrated or more anxious because your guilt has not been relieved*

**Possible sequence of opening events and strength of approach**

**CASE REFERENCES**

1. Park I, Gupta A, Mandani K, et al. “Breaking bad news education for emergency medicine residents: a novel training module using simulation with the SPIKES protocol.” J Emerg Trauma Shock 2010;3:385-388.
2. Nordstrom A, Fjellman-Wiklund A, Grysell T. “The effect of a role-playing exercise on clerkship students’ views of death notification: the Swedish experience.” Int J Med Educ 2011;2:24-29.
3. Bowyer MW, Hanson JL, Pimentel EA, et al. “Teaching breaking bad news using mixed reality simulation.” J Surg Res 2010;159:462-467.
4. Ptacek JT, Eberhardt TL. Breaking bad news: a review of the literature. JAMA 1996;276:496-502.
5. Hobgood C, Tamayo-sarver J, Hollar D, et al. “Griev_Ing: death notification skills and applications for fourth year medical students.” Teach Learn Med 2009;21:207-219.
6. Hobgood C, Harward D, Newton K, et al. The educational intervention “GRIEV_ING” improves the death notification skills of residents.” Acad Emerg Med 2005;12:296-301.

**SPIKES Rating Sheet**

| **Behavior** | **Yes** | **Partially** | **No** |
| --- | --- | --- | --- |
| ***Set the stage*** | | | |
| 1. Clearly introduced herself/himself |  |  |  |
| 1. Clearly stated his/her role in the care of your father (brother) |  |  |  |
| ***Perception*** | | | |
| 1. Determined your level of knowledge of the situation |  |  |  |
| 1. Took note of your vocabulary and framed the conversation in those terms |  |  |  |
| ***Inform*** | | | |
| 1. Briefly outlined the chronology of events leading up to your father (brother)’s death |  |  |  |
| 1. Used plain language, no jargon |  |  |  |
| 1. Avoided euphemisms |  |  |  |
| 1. Delivered a warning shot before telling me my family member died (e.g. "I'm afraid I have some bad news.") |  |  |  |
| ***Knowledge*** | | | |
| 1. Allowed you to react to the information |  |  |  |
| 1. Allowed you to ask questions or express concerns |  |  |  |
| 1. Answered ALL questions in an appropriate manner |  |  |  |
| ***Empathy*** | | | |
| 1. Used proper statements to show concern for your grieving |  |  |  |
| 1. Responded to your emotion through validation, naming, exploration |  |  |  |
| ***Summary and strategy*** | | | |
| 1. Avoided showing any guilt for the loss (“I’m sorry” as the only expression of empathy) |  |  |  |
| 1. Told you how they could be reached to answer questions later or speak to other people |  |  |  |
| 1. Provided closure through summary, making a plan, or asking if there was anything else you need before they leave |  |  |  |

JHU EOL II Death Notification, Singler Case SP Feedback Topics

Student ____________

| Item | **+ Observed behavior/family member reaction –** | |
| --- | --- | --- |
| Providing a warning shot before the actual death notification. |  |  |
| Giving you time to absorb and to speak (looking at you, not rushing to fill any silence.) |  |  |
| Showing empathy (e.g., “I am so sorry that this happened to your wife/husband; I can only imagine how you must be feeling right now.”) |  |  |
| Listening compassionately (nodding, allowing you to speak, maintaining warm eye contact) |  |  |
| Responding to your emotion beyond a surface gesture. |  |  |
| Remaining patient, allowing you to interrupt; answering all questions that you have. |  |  |
| Offering some kind of help: to get you more information, make calls for you, set up a time for the two of you to meet with the resident (if you have made that request). |  |  |
