# Supplementary material for: End-of-Life Care: A Multimodal and Comprehensive Curriculum for Graduating Medical Students Utilizing Experiential Learning Opportunities
Source: MedEdPORTAL. 2021 Apr 27;17:11149. doi: 10.15766/mep_2374-8265.11149 (PMC8076371; doi:10.15766/mep_2374-8265.11149)
Supplement: Supplementary file 1 — End-of-Life 1 Faculty Guide.docxEnd-of-Life 1 Student Handouts.docEnd-of-Life 1 Standardized Patient Materials.docxEnd-of-Life 2 PowerPoint Presentation.pptEnd-of-Life 2 Faculty Guide.docxEnd-of-Life 2 Simulation Materials.docxEnd-of-Life 2 Simulation Case Faculty Guide.docxEnd-of-Life 2 Standardized Patient Materials.docxEnd-of-Life Assessment.docx [file mep_2374-8265.11149-s001.zip › I. End-of-Life Assessment.docx]

**End of Life Module Evaluation**

Questions specific to End of Life 1, 2, or both, are noted in parenthesis after the question

1. Rate the overall quality of this session (End of Life – I)
   1. Poor
   2. Fair
   3. Satisfactory
   4. Very good
   5. Excellent
2. I received clear learning objectives for this session (End of Life – 1)
   1. Strongly disagree
   2. Somewhat disagree
   3. Neither agree nor disagree
   4. Somewhat agree
   5. Strongly agree
3. The session content reflected the learning objectives for the session (End of Life – 1)
   1. Strongly disagree
   2. Somewhat disagree
   3. Neither agree nor disagree
   4. Somewhat agree
   5. Strongly agree
4. List something important that you learned from this session that will help you with internship and residency (End of Life – 1) (open-ended)
5. Please rate this session overall (End of Life – 2)
   1. Poor
   2. Fair
   3. Satisfactory
   4. Very Good
   5. Excellent
6. I received clear learning objectives for this session (End of Life – 2)
   1. Strongly disagree
   2. Somewhat disagree
   3. Neither agree nor disagree
   4. Somewhat agree
   5. Strongly agree
7. The session content reflected the learning objectives (End of Life – 2)
   1. Strongly disagree
   2. Somewhat disagree
   3. Neither agree nor disagree
   4. Somewhat agree
   5. Strongly agree
8. List something important that you learned from this session that will help you with internship and residency (End of Life – 2) (open-ended)
9. The course facilitated the development of lifelong learning habits (End of Life 1 and 2)
   1. Strongly disagree
   2. Somewhat disagree
   3. Neither agree nor disagree
   4. Somewhat agree
   5. Strongly agree
10. Please rate your agreement with the following statements as you think about the transition you are about to make from medical school to internship and residency.
    1. I feel prepared to pronounce a person dead (End of Life 2)
       1. Strongly disagree
       2. Somewhat disagree
       3. Neither agree nor disagree
       4. Somewhat agree
       5. Strongly agree
    2. I feel prepared to correctly complete a death certificate (End of Life 2)
       1. Strongly disagree
       2. Somewhat disagree
       3. Neither agree nor disagree
       4. Somewhat agree
       5. Strongly agree
    3. I feel prepared to discuss advanced care planning, living wills, durable power of attorney, and do not resuscitate orders with patients and families (End of Life 1)
       1. Strongly disagree
       2. Somewhat disagree
       3. Neither agree nor disagree
       4. Somewhat agree
       5. Strongly agree
11. This session provided me with the knowledge and skills to understand the key differences in advanced care planning, living wills, durable power of attorney, and do not resuscitate orders (End of Life 1)
    1. Strongly disagree
    2. Somewhat disagree
    3. Neither agree nor disagree
    4. Somewhat agree
    5. Strongly agree
12. I am able to communicate effectively about advanced care planning and preferences for life-sustaining treatment with a patient (End of Life 1)
    1. Strongly disagree
    2. Somewhat disagree
    3. Neither agree nor disagree
    4. Somewhat agree
    5. Strongly agree
13. I am able to understand the elements of informed consent when communicating with a patient about life-sustaining treatment (End of Life 1)
    1. Strongly disagree
    2. Somewhat disagree
    3. Neither agree nor disagree
    4. Somewhat agree
    5. Strongly agree
14. I feel comfortable explaining the process of pursuing an autopsy to a family member of a deceased patient (End of Life 2)
    1. Strongly disagree
    2. Somewhat disagree
    3. Neither agree nor disagree
    4. Somewhat agree
    5. Strongly agree
15. I feel confident in my ability to accurately complete a death certificate (End of Life 2)
    1. Strongly disagree
    2. Somewhat disagree
    3. Neither agree nor disagree
    4. Somewhat agree
    5. Strongly agree
16. I understand my role as a physician in the process of organ donation (End of Life 2)
    1. Strongly disagree
    2. Somewhat disagree
    3. Neither agree nor disagree
    4. Somewhat agree
    5. Strongly agree
17. I have a better understanding of how to decide when it is appropriate to stop resuscitation efforts (End of Life 2)
    1. Strongly disagree
    2. Somewhat disagree
    3. Neither agree nor disagree
    4. Somewhat agree
    5. Strongly agree
18. I feel comfortable informing a family member about the death of a loved one (End of Life 2)
    1. Strongly disagree
    2. Somewhat disagree
    3. Neither agree nor disagree
    4. Somewhat agree
    5. Strongly agree
19. Rate the overall performance of the instructors (End of Life 1 and 2)
    1. Poor
    2. Fair
    3. Average
    4. Good
    5. Exceptional
