# Supplementary material for: End-of-Life Care: A Multimodal and Comprehensive Curriculum for Graduating Medical Students Utilizing Experiential Learning Opportunities
Source: MedEdPORTAL. 2021 Apr 27;17:11149. doi: 10.15766/mep_2374-8265.11149 (PMC8076371; doi:10.15766/mep_2374-8265.11149)
Supplement: Supplementary file 1 — End-of-Life 1 Faculty Guide.docxEnd-of-Life 1 Student Handouts.docEnd-of-Life 1 Standardized Patient Materials.docxEnd-of-Life 2 PowerPoint Presentation.pptEnd-of-Life 2 Faculty Guide.docxEnd-of-Life 2 Simulation Materials.docxEnd-of-Life 2 Simulation Case Faculty Guide.docxEnd-of-Life 2 Standardized Patient Materials.docxEnd-of-Life Assessment.docx [file mep_2374-8265.11149-s001.zip › B. End-of-Life 1 Student Handouts.doc]

# ADULT CASE SUMMARY

# Life-Sustaining Treatment: Ben/Benita Ward

**Student Handout**

**OVERVIEW**

**Objective:**

To enhance fourth year medical students’ skills in discussing goals and preferences for life-sustaining treatment with a hospitalized patient.

**Competencies Assessed:**

1. Patient education and counseling skills

2. Negotiation and shared decision-making skills

3. Relationship development skills

4. Conflict resolution skills

5. Global satisfaction

**STUDENT INSTRUCTIONS**

**Patient Information: Ben/Benita Ward**

**Your Role:**

You are a medicine intern. You are on call and admitted a patient with a chronic obstructive pulmonary disease (COPD) exacerbation. While presenting the case to the senior resident, she asks you about the patient’s “code status.” You have not had that conversation with the patient yet, so you have to go back and discuss it with them. It’s been a busy day and you only had about 30 minutes to spend with Mr./Ms. Ward previously.

**Situation:**

Mr./Ms. Ward is a 65-year-old with moderate-severe COPD who was admitted for the 2^nd^ time in nine months with a COPD exacerbation. This is the patient’s fifth admission overall for COPD. He/she developed worsening shortness of breath, and increased volume of sputum production over the past four days. History, exam, and chest radiograph are consistent with a COPD exacerbation. You started the patient on methylprednisolone, doxycycline, and bronchodilators. He/she is starting to feel better.

Mr./Ms. Ward first developed dyspnea on exertion (DOE) about 10 years ago and was diagnosed with COPD. He/she smoked two packs per day for 45 years but quit two years ago. The patient reports that their DOE has worsened every year. They can walk up a flight of stairs but is very winded at the top and stops to rest and coughs daily. The patient does not require oxygen. They have continued to work, but it is getting harder to do so.

You think Mr./Ms. Ward will turn around in the next 48 hours. However, last month you had a patient who came in with a congestive heart failure exacerbation. You thought the same thing about her, but then she had a cardiac arrest and underwent cardiopulmonary resuscitation (CPR) before you could talk to her about her preferences. She survived but spent weeks in the intensive care unit (ICU) and suffered hypoxic encephalopathy.

Mr./Ms. Ward has an forced expiratory volume_1_ (FEV1) that is 30% of predicted value and has a body mass index greater than 21. If asked, would rate themself with mild-moderate dyspnea on validated scales, and has somewhat limited ability to walk a long distance in six minutes. He/she has never been intubated and does not require chronic steroids. While it is difficult to predict mortality in COPD, based on some prognostic models, his/her risk of death from any cause in the next 3 years is 10-25%.

You know that if Mr./Ms. Ward has a cardiac arrest in the hospital, his/her chance of surviving to discharge is about 15% and the chance of being alive at 12 months is about 10%. Given his/her COPD, it is less clear, if intubated, whether he/she would be able to come off the ventilator. Other than death, there are other significant risks associated with mechanical ventilation and CPR, including debility/functional impairment that does not improve, brain damage, and a host of other medical complications (infection, pressure ulcers, etc.).

PMH: COPD (FEV1 0.9L), hypertension

Home Meds: tiotropium, fluticasone, albuterol, hydrochlorothiazide

Records from the patient’s primary care physician indicate they were concerned about his/her oxygen saturation with exercise, even when at baseline, and there are no documented advance directives.

Family Hx: both parents died from myocardial infarctions

Social Hx: married, lives with spouse in Arbutus. Has two children and two grandchildren. Works as a delivery person for a linen service that supplies local hospitals and nursing homes. Completed the 11^th^ grade. Quit smoking two years ago.

**Your Task:**

Discuss his/her “code status” or preferences for life sustaining treatment.

**Further Readings:**

1. Briggs A, Spencer M, Wang H, et al. Development and Validation of a Prognostic Index for Health Outcomes in Chronic Obstructive Pulmonary Disease. Arch Intern Med. 2008;168(1):71-79.

2. Celli B, Cote C, Marin J, et al. The Body-Mass Index, Airflow Obstruction,

Dyspnea, and Exercise Capacity Index in Chronic Obstructive Pulmonary Disease. N Engl J Med 2004;350:1005-12.

3. Dolan S, Varkey B. Prognostic factors in chronic obstructive pulmonary disease. Curr Opin Pulm Med 2005;11:149-152.

4. Tulsky JA. “Beyond advance directives: Importance of communication skills at the end of life.” JAMA 2005;294:359-65.

5. Roter DL, et al. “Experts practice what they preach.” Arch Intern Med 2000;160:3477-85.

6. Quill TE. “Initiating end-of-life discussions with seriously ill patients.” JAMA 2000;284: 2502-7.

7. Tulsky JA, et al. “Opening the black box: How do physicians communicate about advance directives?” Ann Intern Med 1998;129:441-9.

8. Tulsky JA, et al. “How do medical residents discuss resuscitation with patients?” J Gen Intern Med 1995;10:436-42.

9. Cooper S, Janghorbani M, Cooper G, et al. A decade of in-hospital resuscitation: outcomes and prediction of survival. Resuscitation 2006;68:231-237.

# PEDIATRIC CASE SUMMARY

# Life-Sustaining Treatment: Kelly Ward (parent: Sam Ward)

**Student Handout**

**OVERVIEW**

**Objective:**

To enhance fourth year medical students’ skills in discussing goals and preferences for life-sustaining treatment with the parent of a terminally ill, hospitalized child.

**Competencies Assessed:**

1. Patient education and counseling skills

2. Negotiation and shared decision-making skills

3. Relationship development skills

4. Conflict resolution skills

5. Global satisfaction

**INSTRUCTIONS FOR THE STUDENT**

**Patient Information: Kelly Ward**

**Your Role:**

You are a pediatrics intern doing a rotation in the pediatric intensive care unit (PICU). A 9-year-old girl was admitted two days ago with an intracranial hemorrhage. You just rotated onto the service and are on rounds this morning, the team discussed the fact that she had not made any progress towards recovery. There was concern that a) she may deteriorate at any time, including herniation or having a possible cardiac arrest and b) resuscitation attempts were not likely to restore quality of life. The patient’s oncologist is out of town. The PICU attending asks if you would like to lead the discussion (with them present) with the patient’s father about whether or not cardiopulmonary resuscitation (CPR) would be appropriate or desired.

**Situation:**

Kelly Ward is a 9-year-old girl diagnosed with a brain tumor (glioblastoma) eight months ago. She underwent surgical resection but unfortunately, the entire tumor could not be removed. She subsequently had a ventriculoperitoneal shunt placed for obstructive hydrocephalus.

Because the entire tumor could not be removed, Kelly has been undergoing chemotherapy and radiation, for almost the entire last seven months. This requires her to go to the hospital several times per week for treatment. She has been hospitalized twice since the initial surgery, both times for infections (pneumonia). She has never been intubated before (until this hospitalization).

Two days ago, while playing on her handheld gaming device, she had the acute onset of dysarthria, confusion, and the inability to stand. She was immediately brought to the hospital. Neuroimaging showed an acute hemorrhage in and around the tumor causing mild midline shift and ipsilateral ventricular compression. She is now in the PICU and intubated for airway protection. She is still lethargic but responds to voice and stimulation by attempting to open her eyes.

Over the past 48 hours, Kelly has not shown any signs of improvement and has had progressive deterioration of her Glasgow Coma Scale (GCS). She is being treated with supportive care, including medical measures to decrease her increased intracranial pressure and intensive neuromonitoring, but the PICU physicians believe that she could herniate at any time. The neurosurgeons who have consulted do not think any further surgical intervention would be of benefit at this time.

Before this event, her prognosis was poor given the aggressive nature of her tumor.

The chances of her surviving this event and returning to her previous quality of life are almost nonexistent given the extent of the bleeding.

Past Medical History: None other than stated above. Normal development, normal vaccination schedule.

Social History: Lives with her father (Sam Ward). Her mother died two years ago in a motor vehicle collision. Attends 3^rd^ grade.

**Your Task:**

Discuss Kelly’s father’s wishes about CPR. You are not being asked to discuss removing the ventilator at this time. He was told by the team previously the medical facts to date.

**Further Readings:**

1. Berkowitz I, Morrison W. “Do not attempt resuscitation orders in pediatrics.” Pediatr Clin N Am 54 (2007) 757–771.
2. Hinds P, et al. “Trying to be a good parent” as defined by interviews with parents who made phase I, terminal care, and resuscitation decisions for their children.” J Clin Oncol 27:5979-5985.
3. Tulsky JA. “Beyond advance directives: Importance of communication skills at the end of life.” JAMA 2005;294:359-65.
4. Roter DL, et al. “Experts practice what they preach.” Arch Intern Med 2000;160:3477-85.
5. Quill TE. “Initiating end-of-life discussions with seriously ill patients.” JAMA 2000;284: 2502-7.
6. Tulsky JA, et al. “Opening the black box: How do physicians communicate about advance directives?” Ann Intern Med 1998;129:441-9.
7. Tulsky JA, et al. “How do medical residents discuss resuscitation with patients?” J Gen Intern Med 1995;10:436-42.
